# Controlling the Glycosylation Profile in mAbs Using Time-Dependent Media Supplementation

**DOI:** 10.3390/antib7010001

**Published:** 2017-12-21

**Authors:** Devesh Radhakrishnan, Anne S. Robinson, Babatunde A. Ogunnaike

**Affiliations:** 1 Department of Chemical and Biomolecular Engineering, University of Delaware, Newark, DE 19716, USA; devesh@udel.edu; 2 Department of Chemical and Biomolecular Engineering, Tulane University, New Orleans, LA 70118, USA; asr@tulane.edu

**Keywords:** cell culture, glycosylation, media supplementation, MnCl_2_, controllability analysis

## Abstract

In order to meet desired drug product quality targets, the glycosylation profile of biotherapeutics such as monoclonal antibodies (mAbs) must be maintained consistently during manufacturing. Achieving consistent glycan distribution profiles requires identifying factors that influence glycosylation, and manipulating them appropriately via well-designed control strategies. Now, the cell culture media supplement, MnCl_2_, is known to alter the glycosylation profile in mAbs generally, but its effect, particularly when introduced at different stages during cell growth, has yet to be investigated and quantified. In this study, we evaluate the effect of time-dependent addition of MnCl_2_ on the glycan profile quantitatively, using factorial design experiments. Our results show that MnCl_2_ addition during the lag and exponential phases affects the glycan profile significantly more than stationary phase supplementation does. Also, using a novel computational technique, we identify various combinations of glycan species that are affected by this dynamic media supplementation scheme, and quantify the effects mathematically. Our experiments demonstrate the importance of taking into consideration the time of addition of these trace supplements, not just their concentrations, and our computational analysis provides insight into what supplements to add, when, and how much, in order to induce desired changes.

## 1. Introduction

The global market for pharmaceuticals is predicted to grow to $1.5 trillion by 2021, with biologics such as monoclonal antibodies (mAbs), hormones, and therapeutic enzymes accounting for nearly 20% of the market share, based on current projections. With US sales rising from $8.29 billion in 2005 to $24.6 billion in 2012 [1,2]—coupled with the increase in regulatory agencies approvals of mAb treatments for different indications ranging from cancer to rheumatoid arthritis and Crohn’s disease [3,4], the growing market for mAbs has resulted in active development of these biological products. While several different mammalian cell expression systems can be used to synthesize mAb therapeutics, over half of all currently approved mAbs are produced in Chinese Hamster Ovary (CHO) cell lines. CHO cells are the preferred host for a large number of recombinant mAb therapeutics because of the ease of adapting them to suspension growth, and the availability of powerful gene amplification systems to improve specific productivity [5]. However, more importantly, the post-translational modification machinery in CHO cells produces human-like structures in mAbs, thus ensuring biocompatibility. 

One important post-translational modification in mAbs is N-glycosylation, a process by which an oligosaccharyltransferase complex in the endoplasmic reticulum adds a sugar substrate (glycan) to the Asn-X-Ser/Thr motif in the heavy chain of the mAb (where X is any amino acid other than Pro). As the mAb traverses the Golgi complex, the core glycan undergoes a series of non-template driven reactions that are mediated by the localized glycosyltransferase enzymes in the different Golgi compartments [6,7]. The result is a heterogeneous distribution of glycan residues or glycoforms, which affects the immunogenicity, effector functions, and the pharmacokinetic properties of the mAb, and consequently the final drug product quality [8,9,10]. Thus, there is considerable motivation for manufacturers and regulators to understand, characterize, and, if necessary, modulate the glycoform distribution in mAbs during production, in order to maintain a consistent glycan profile [11,12,13,14]. However, manipulating the glycan distribution effectively requires (i) identifying the factors that can influence the glycan distribution; (ii) quantifying the degree to which these factors affect the concentration of the glycoform species; and (iii) using such information to design effective control systems. 

Previous studies have demonstrated that protein glycosylation can be influenced by various factors [15], such as pH [16,17], temperature [18,19,20], dissolved oxygen [21,22], ammonia [23], and media supplements such as nucleotide sugar precursors [24] and manganese chloride (MnCl_2_) [25,26,27,28]. Each study focused on an individual factor, establishing empirical relationships between the individual factor in question and the specific set of glycan species it affects. However, modulating the complete glycan distribution profile requires manipulating multiple input factors simultaneously, and to be effective, such action must be based on a thorough, holistic understanding of how these inputs individually and jointly affect various glycan species. Such process understanding can be generated systematically by using statistical design of experiments whereby input factors are judiciously varied simultaneously to generate data on the main and interaction effects they exert on all the output responses of interest. Such structural information indicates which inputs to manipulate, and by how much, in order to alter the relative concentrations of different glycan species appropriately. In most cases, however, the available inputs are fewer than the glycan species to be controlled, resulting in a system with insufficient degrees of freedom. Consequently, we must first answer a fundamental question: given a limited set of inputs, to what extent can we independently control the concentrations of all the desired glycan species? In other words, is the desired change in the glycan distribution achievable using the available inputs? We address this question using “controllability analysis”, by which we can determine quantitatively the extent to which the system is controllable. (Informally, a system is considered completely controllable if it is possible to drive the complete set of outputs from some initial value to any arbitrarily specified final, desired value, by manipulating the available set of inputs.) We have previously introduced, in [29], the concept of output controllability, demonstrated how to use it to assess the controllability of the glycan reaction network using data generated from statistical design of experiments, and in [30] we illustrated practical applicability by using it to identify in an experimental system, the glycan species whose concentrations can be controlled using such media supplements as MnCl_2_, galactose and NH_4_Cl as manipulated variables. 

The role of different media supplements in modulating critical quality attributes of the mAb in general, and the glycan distribution profile in particular, has received considerable attention recently [31]. Typically, supplements such as MnCl_2_, which are known to affect the expression and activity of several glycosyltransferase enzymes, are added to the media at the start of the batch to alter the glycan distribution. However, over the course of the batch run, as the cells continue to grow and produce mAb molecules, it is clear that changes in the cellular availability of supplements will influence not just the antibody productivity but also the activity of the glycosyltransferase enzymes, thus affecting the final glycan distribution. Consequently, we postulate that it is possible to control the glycosylation profile in mAbs by introducing specific media supplements at different stages of cell growth. We postulate further that introducing a chelating agent to the media can alter the effect of MnCl_2_ addition on the glycan distribution. Consequently, a chelating agent such as EDTA provides an additional degree of freedom for fine-tuning the effect of MnCl_2,_ the media supplement of primary interest. Although EDTA is toxic to cells and can titrate both Mn^++^ and other bivalent ions in the medium that are necessary for cell growth and may be cofactors for other glycosyltransferase enzymes, it has been used in this study primarily to elucidate the effects of a dynamic addition of the two media supplements. (It is important to stress that the proposed time-dependent media supplementation is distinct from current fed-batch strategies where the objective is primarily to meet the nutrient demand in the culture, for the express purpose of enhancing cell growth and productivity—not to ensure that the product quality meets specific targets.) Specifically, we aim to identify the glycan species that can be controlled by adding MnCl_2_ during lag, exponential, and stationary phases of cell growth, and to quantify the effect of such time-dependent MnCl_2_ additions on the glycan distribution.

In this work, we use a mixed factorial experimental design to add MnCl_2_ and EDTA at various stages of cell growth and, via appropriate analysis of the resulting data, we quantify the effect of time-dependent media supplementation on the antibody productivity and the glycosylation profile in mAbs. Subsequently, we use controllability analysis to identify the glycan species whose relative percentages can be controlled effectively by introducing MnCl_2_ and EDTA to the media at different time points, and quantify the effect of these time-dependent additions. Overall, our results highlight the importance of taking into account the dynamic nature of media supplementation, and also provide concepts that can be exploited to develop new strategies for controlling the glycosylation profile in mAbs. 

## 2. Materials and Methods

### 2.1. Cell Culture 

All experiments were conducted using an IgG1 producing CHO-K1 cell line donated by Genentech, San Francisco, California. The cells were scaled up in a custom CD OptiCHO^TM^ medium formulation (Thermo Fisher Scientific, Waltham, MA, USA) that was supplemented with 4 mM glutamine, 5 g/L glucose and 25 nM MTX. The osmolality was adjusted to 300 mOsm by adding NaCl stock solution. The concentration of MnCl_2_ in the media was adjusted using a 0.5 M stock solution (Sigma Aldrich, St. Louis, MO, USA). Similarly, a 0.5 M EDTA sterile stock solution was prepared and added to the media as required. The cells were inoculated with an initial seeding density of 0.5 × 10^6^ cells/mL in vented-cap Erlenmeyer shake flasks with a working volume of 50 mL and grown in batch in suspension in an incubator maintained at 37 °C with a 5% CO_2_ overlay, with supplements only as indicated by experimental design below. Cell count measurements were taken every two days using a hemocytometer. Metabolite (glutamine, glucose, glutamate and lactate) concentrations, media pH, and osmolality were measured using a Bioprofile 100+ analyzer (Nova Biomedical, Waltham, MA, USA). Antibody titer was measured with an Agilent 1200 HPLC instrument using 1X PBS buffer on a Thermo Scientific^TM^ MAbPac Protein A chromatography column (12 micron particle size, 35 × 4.0 mm I.D., Thermo Fisher Scientific, Waltham, MA, USA). 

### 2.2. Experimental Design

The shake flask experiments were conducted according to a (2^2^, 3^2^) mixed level experimental design for the following factors: (i) MnCl_2_ concentration (high and low levels); (ii) EDTA concentration (high and low levels); (iii) time of addition of MnCl_2_ (high, intermediate, and low levels); and (iv) time of addition of EDTA (high, intermediate and low levels). The concentration of MnCl_2_ in the basal media corresponds to the low-level condition (−1) for MnCl_2_, while the high-level condition (+1) corresponds to the final concentration of MnCl_2_ supplemented media (0.04 mM). High and low levels for MnCl_2_ were-based upon previous experiments performed using the same cell line [30]. Similarly, the low-level condition (−1) for EDTA corresponds to “no EDTA” added to the media, while the high level (+1) corresponds to 0.08 mM EDTA added to the media. MnCl_2_ and EDTA are added on day 0 (D0), day 3 (D3), or day 6 (D6) after inoculation, corresponding respectively to the low (−1), intermediate (0), and high (+1) levels. Thus, this full factorial mixed level (2^2^, 3^2^) experimental design yields a total of 36 different possible shake flask conditions to be tested. However, the 36 conditions are not unique because some of the cases correspond to identical experimental conditions. For instance, 9 of the 36 conditions correspond to MnCl_2_ and EDTA at low levels (−1), with the time of addition at low (−1), intermediate (0) and high levels (+1). The low level condition for MnCl_2_ represents basal concentrations, while the low level for EDTA represents no EDTA supplementation. Thus, these 9 cases represent identical conditions where the flask has basal levels of MnCl_2_ with no EDTA supplementation either on D0, D3, or D6. A single flask (F1) was used for all nine cases and was treated as the control flask because of what the conditions represent—basal level of MnCl_2_ and no EDTA supplementation on any day. It can be shown that there are in fact only 16 unique experimental cases/conditions, as listed in Table 1. Each condition was tested with two biological replicates. The glycan distribution profile was determined using the permethylation assay described below, and the resulting relative glycan percentages data obtained for each condition were analyzed in MINITAB using standard analysis of variance (ANOVA) to obtain the factor effects/coefficients and associated *p*-values. 

### 2.3. Glycan Permethylation Assay

On day 8 after inoculation, the cells were centrifuged at 3000 rpm for 10 min and the spent media was harvested. The IgG1 antibody was then purified from the spent media using a PhyNexus Benchtop MEA2 system using Protein A chromatography resin packed in a 2 mL PhyTip column (PhyNexus, San Jose, CA, USA). The glycan permethylation assay was then carried out with 100 microgram of the purified antibody using a previously described method [30]. Briefly, the antibody was first digested with trypsin (Promega, Madison, WI, USA) for four hours in an incubator held at 37 °C, followed by enzymatic deglycosylation using PNGase-F (ProZyme, Hayward, CA, USA) for a minimum of 16 h at 37 °C. The free separated glycans were captured on Hypersep Hyper Carb SPE cartridges (Thermo Fisher Scientific, Waltham, MA, USA) and permethylated following the Ciucanu method using methyl iodide and NaOH in the presence of DMSO [32,33]. The permethylated glycans were purified in a liquid-liquid extraction step with chloroform (Sigma Aldrich, St. Louis, MO, USA), dried and resuspended in 80% methanol (Sigma Aldrich, St. Louis, MO, USA). The resuspended glycans were spotted onto a MALDI/TOF plate with a DHB matrix and analyzed using a 4800 MALDI TOF/TOF Analyzer (ABSciex) in positive ion, reflector mode. The data collected using the mass spectrometer was then exported to DataExplorer to obtain the peak heights for the identified glycans (see Supplementary Table S8). The relative glycan distribution in each sample was calculated from the sum of the peak heights for all the identified glycans in that sample.

### 2.4. Glycosylation Index

For each experimental condition, glycosylation indices were calculated from the relative percentages of individual glycan species [17,34]. For example, the galactosylation index (GI), defined as the percentage of mono- and di-galactosylated species in the total glycan distribution, was determined according to Equation (1):

(1) $$\mathrm{GI}=\frac{2\times G_{2}+G_{1}}{2\times \left(G_{0}+G_{1}+G_{2}\right)}\%,$$

where G_0_ is the sum of all agalactosylated species, G_1_ is the sum of all monogalactosylated species, and G_2_ is the sum of all digalactosylated species. Similarly, we calculated the fucosylation index (FI) for each distribution as

(2) $$\mathrm{FI}=\frac{F_{1}}{\left(F_{0}+F_{1}\right)}\%,$$

where F_0_ and F_1_ are the sum of all afucosylated and fucosylated species, respectively. 

### 2.5. Controllability Analysis

Using the technique presented in St.Amand et al. [29], we performed controllability analysis to quantify the effect of time dependent media supplementation on the glycosylation profile in mAbs. Briefly, we note first that estimates of factor coefficients obtained after analyzing the mixed factorial design data correspond to the various “process gains”, defined as the change observed in the glycan distribution (output), **∆y**, in response to a unit change in the input factor with which the coefficient in question is associated. By selecting statistically significant factor coefficients (at the significance level of α = 0.05) and setting all non-significant coefficients to zero, we generateed the process gain matrix **K** so that:

(3) Δy=KΔu,

where **∆u** represents the change in the input factor. Singular value decomposition of the process gain matrix produces the diagonal singular value matrix, **Σ**, and the unitary matrices, **W** and **V^T^**, that are subsequently used to obtain the orthogonal input (**μ**) and output (**η**) modes, which, along with the corresponding singular values are used to assess controllability. 

## 3. Results

### 3.1. Early Addition of EDTA Was Detrimental to Cell Growth and Reduces Antibody Titer

**Figure 1:**
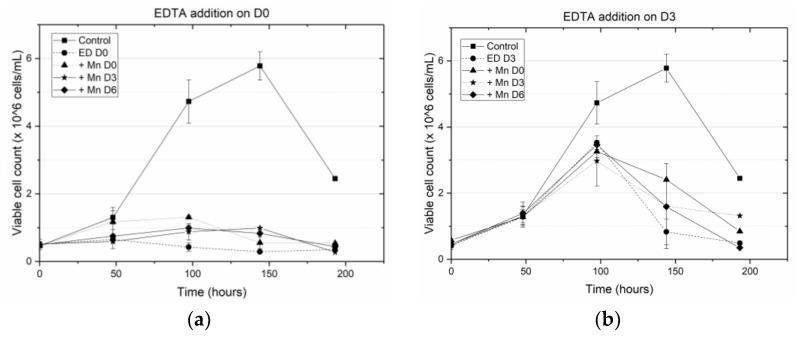

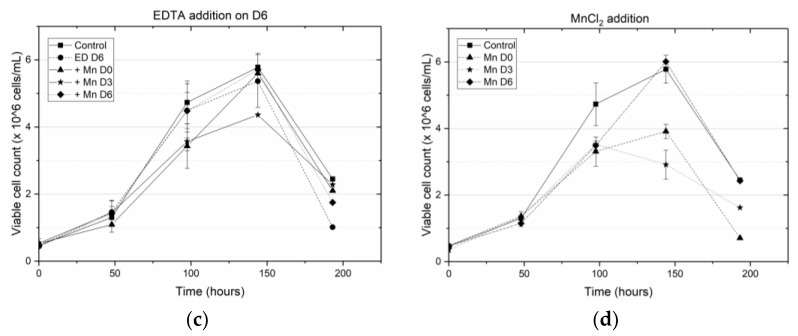
Average viable cell concentration data for CHO-K1 cells when (**a**) EDTA is added by itself on D0 (●), or in the presence of MnCl_2_ on D0 (▲), D3 (*), and D6 (♦); (**b**) EDTA is added by itself on D3 (●), or in the presence of MnCl_2_ on D0 (▲), D3 (*), and D6 (♦); (**c**) EDTA is added by itself on D6 (●), or in the presence of MnCl_2_ on D0 (▲), D3 (*), and D6 (♦); and (**d**) MnCl_2_ is added on D0 (▲), D3 (*), and D6 (♦).

**Figure 2:**
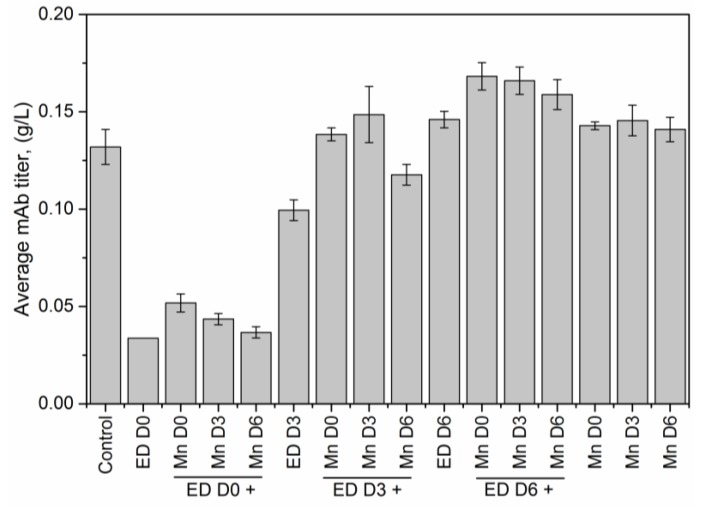
Average antibody titer for the 16 experimental conditions. Mn and ED refer to the media supplements MnCl_2_ and EDTA, while D0, D3 and D6 refer to Day 0, Day 3 and Day 6 after inoculation, respectively. Error bars represent the range of biological replicates (*n* = 2).

Figure 1 and Figure 2 show the effect that introducing media supplements (MnCl_2_ or EDTA) at different time points had on viable cell density and final antibody titer, compared to corresponding results obtained from a control flask (F1), which contained MnCl_2_ at basal media concentrations and no EDTA (see Figure S2 in supplementary information for the normalized glucose concentration in each shake flask). 

Compared to the conditions in the control flask, early addition of EDTA on D0 reduced the cell density significantly, which is consistent with hampered cell growth. However, this effect was offset somewhat by introducing MnCl_2_ in addition to EDTA on D0. The average viable cell density (VCD) measured on day 4 for samples in which both MnCl_2_ and EDTA were introduced on D0, was 1.31 × 10^9^ cells/L, which was nearly three times as large as the value of the VCD in the flasks where only EDTA was added on D0 (~0.4 × 10^9^ cells/L). Introducing MnCl_2_ on D3 or D6 after the addition of EDTA on D0 did not improve the VCD. Similarly, when EDTA was introduced on D3, the VCD in samples with no additional MnCl_2_ supplementation dropped sharply. By contrast, when the media was supplemented with MnCl_2_ on D0 or D3, the VCD was slightly higher than when the media was supplemented with just EDTA on D3. Supplementing the samples with MnCl_2_ on D6 after the addition of EDTA did not improve the VCD significantly. In all cases, the observed VCD was generally higher than when EDTA was introduced on D0. Addition of EDTA on D6 had no impact on the VCD, regardless of the time of introduction of MnCl_2_. Similarly, as shown in Figure 1d, early addition of MnCl_2_ on D0 and D3 reduced the VCD, while addition of MnCl_2_ on D6 did not alter the VCD. Thus, in summary, early addition of EDTA reduced cell viability in the absence of MnCl_2_ supplementation, but the addition of MnCl_2_ by itself did not alter cell viability significantly.

Figure 2 shows the effect of media supplementation on antibody titers. The average mAb titer in the control flask F1 (with no MnCl_2_ or EDTA supplementation) was 0.13 g/L. The addition of EDTA to the media on D0 in the absence of MnCl_2_ supplementation decreased the titer by about a fourth, to 0.03 g/L. This decrease in the titer was marginally offset when MnCl_2_ was introduced on D0, D3, or D6, with earlier MnCl_2_ supplementation resulting in higher titers than later supplementation. When EDTA was introduced on D3, the resulting titer was 0.10 g/L, three times higher than that observed with EDTA supplementation on D0. Further supplementing the media with MnCl_2_ on D0, D3, or D6, increased the titer observed with EDTA supplementation on D3 to values comparable to that of the control case. Supplementing the flasks on D6 with EDTA increased titers even further to 0.15 g/L, beyond values obtained in the control case. The titer values were also higher when EDTA supplementation on D6 was combined with MnCl_2_ supplementation on D0 (0.17 g/L), D3 (0.17 g/L) or D6 (0.16 g/L). Finally, MnCl_2_ supplementation alone on D0, D3, or D6 increased the titer to an average of 0.14 g/L. Thus, while early EDTA supplementation has an adverse effect on the antibody titer, late EDTA addition improves the final titer. Consequently, we conclude that early addition of EDTA reduces antibody titer, while latter addition of EDTA helps to increase the titer by a marginal amount.

### 3.2. Early Addition of MnCl_2_ Alters the Glycan Distribution Significantly

**Figure 3:**
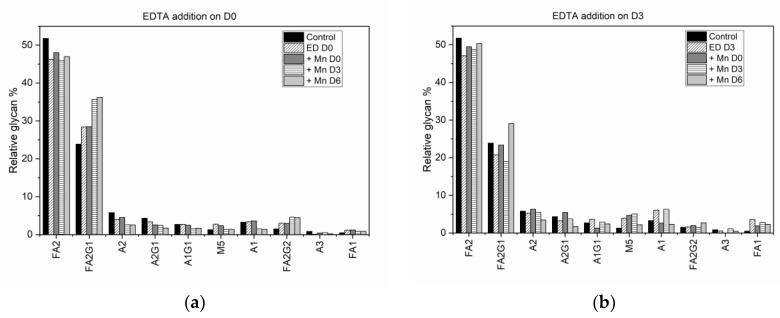

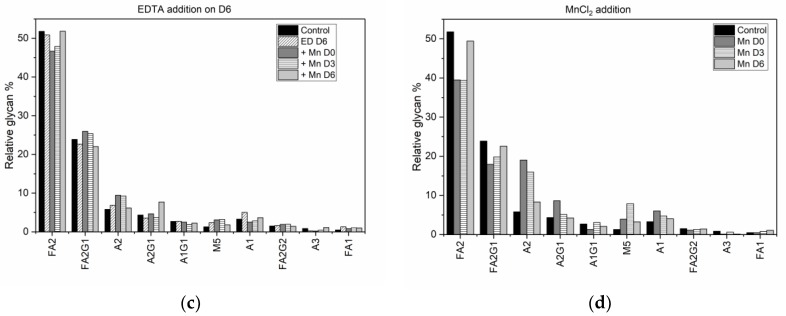
Average relative glycan percentage of select IgG1 glycans produced in CHO-K1 cells when: (**a**) EDTA is added on D0 with no MnCl_2_ supplementation or with MnCl_2_ supplementation on D0, D3, and D6; (**b**) EDTA added on D3 with no MnCl_2_ supplementation or with MnCl_2_ supplementation on D0, D3, and D6; (**c**) EDTA added on D6 with no MnCl_2_ supplementation or with MnCl_2_ supplementation on D0, D3, and D6; and (**d**) MnCl_2_ is added on D0, D3, and D6.

The addition of EDTA and MnCl_2_ at different time points altered the glycan distribution, with earlier addition of MnCl_2_ having the more significant effect. Figure 3 shows the effect of media supplementation on select glycan species, with panels 3(a), (b) and (c) showing the changes in the glycan profile as a result of media supplementation with EDTA on D0, D3, and D6, with or without additional MnCl_2_ supplementation, and 3(d) showing the impact of MnCl_2_ addition in the absence of EDTA supplementation. (See Supplementary Information Figure S3 for the average relative glycan percentage of all the glycan species.)

Adding EDTA to the cell culture media on D0 (Figure 3a) decreased the amount of biantennary fucosylated species, FA2, by 5.58%, which was offset by a concomitant increase in its galactosylated isoforms, FA2G1 (4.52%) and FA2G2 (1.52%). (See supplementary information Table S1 for a complete list of experimentally observed glycan species and their structures.) The mannosylated species M5 also increased by 1.52%, with a corresponding reduction of 1.79% in the concentration of the biantennary group A2 (which is produced from M5 in the Golgi compartment). Adding MnCl_2_ on D0, D3, or D6 to media in which EDTA was introduced on D0 resulted in a further increase in the relative concentrations of the galactosylated isoforms FA2G1 and FA2G2, in addition to a decrease in the relative percentage of FA2, A2, and A2G1 species, compared to the distribution in the control flask. However, the increase in FA2G1 and FA2G2 was more pronounced when MnCl_2_ was introduced on D3 or D6 after adding EDTA. When EDTA was added on D3 (Figure 3b) the relative percentages of the biantennary species FA2, FA2G1, and A2G1 decreased, while the relative percentages of glycan species M5, A1, and FA1 increased. When MnCl_2_ was added on D0 followed by EDTA supplementation on D3, the concentrations of M5 and FA1 increased, while the concentration of FA2 decreased. Additionally, there was an increase in the relative percentage of A2G1 that was offset by the decrease in the concentration of A1G1. When both EDTA and MnCl_2_ were added to the media on D3, the change in the glycan distribution mirrors the trend observed when only EDTA was introduced on D3. The main difference occurs when MnCl_2_ was added to the media after EDTA addition, i.e., on D6. Here, the relative percentages of M5, FA1, FA2G1, and FA2G2 increased while the relative percentages of A2, A2G1, and FA2 decreased, similar to the trends observed with the addition of MnCl_2_ on D3 or D6 following EDTA supplementation on D0. Although the late addition of EDTA on D6 of the cell culture did not affect the glycan profile significantly (Figure 3c), for those glycan species whose relative percentages change, the trend was similar to that observed with EDTA supplementation on D0 and D3, i.e., an increase in the relative percentage of M5 and A1 accompanied by a decrease in that of FA2 and FA2G1. When the media was first supplemented with MnCl_2_ on D0 or D3, followed by EDTA supplementation on D6, the relative percentage of FA2 species decreased while the relative percentages of M5, A2 and FA2G1 species increased. A simultaneous addition of both EDTA and MnCl_2_ toward the end of the batch increased the relative percentage of A2G1 while decreasing the relative amount of its fucosylated isoform FA2G1. 

The solo effect of MnCl_2_ on the glycan profile was observed by supplementing the media with MnCl_2_ in the absence of EDTA on D0, D3 or D6 (Figure 3d). Adding MnCl_2_ on D0 decreased the relative percentage of the FA2 species from the control value of 51.79% (with a 95% confidence interval range of ±0.94%) to 39.50% (±1.55%). Similarly, the relative percentage of the monogalactosylated species FA2G1 dropped from the control value of 23.87% (±0.94%) to 17.95% (±1.55%). This decrease in the fucosylated species was offset by an increase in the biantennary species A2 (which increased by 13.24%), the mannosylated species M5 (2.62%), and the galactosylated biantennary species A2G1 (4.29%). It is important to note that this trend (observed in the glycan distribution when only MnCl2 is added to the shake flask) is consistent with the trend previously observed in experiments performed using the same cell line [30]. Introducing MnCl_2_ to the culture medium on D3 resulted in a similar trend, with the decrease in the fucosylated species being offset by a significant increase in the biantennary species A2 (which increased by 10.20%), the mannosylated species M5 (increasing by 6.59%) and only a marginal increase in the A2G1 species (by 0.76%). Adding MnCl_2_ during the peak exponential phase (D6) produced similar changes in the glycan distribution, but these changes were smaller in comparison to the changes in the glycan distribution due to earlier addition of MnCl_2_. The concentrations of the fucosylated species FA2 and FA2G1 decreased by 2.36% and 1.28% respectively, with the corresponding increases in the concentrations of A2 and M5 species being 2.52% and 1.94%, respectively. 

While such qualitative discussions of the glycan distribution profiles may be instructive in general, for our purposes in this work, a quantitative analysis relating the changes in the experimental conditions to the observed changes in the glycan profile is more informative.

### 3.3. The Type of Media Supplement and the Time of Addition both Showed Statistically Significant Effects on Glycan Distribution

**Figure 4:**
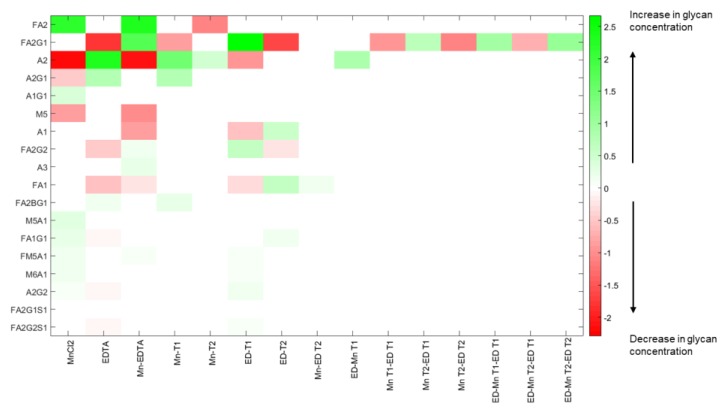
Heat map of significant factor coefficients (α = 0.05, see Table S3 for details) obtained from experimental data analysis using ANOVA. The input factors are on the horizontal axis, and individual glycan species on the vertical axis. The color red indicates a decrease in the concentration of a particular glycan in response to a positive change on the input factor in questions, while green indicates an increase. The color intensity represents the magnitude of the significant coefficient, with increasingly darker hues indicating increasingly larger magnitudes and progressively lighter hues indicating commensurately lower magnitudes.

The concentrations of MnCl_2_ and EDTA and the time of addition of each one constitute the four factors in the (2^2^, 3^2^) mixed factorial experimental design used in this study. In general, the full complement of this factorial experimental design consists of 4 × 9 = 36 independent experimental conditions, and the resulting model, in principle, consists of 35 main and interaction effects. The statistically significant factor coefficients (at the significance level of α = 0.05) estimated using ANOVA, were used to generate a “gain” matrix (see Table S3 in Supplementary Information), whose elements represent “by how much” each output variable (relative glycan distribution) will change in response to a unit change in each input factor, including multi-factor interactions (since multiple combinations of single inputs are considered as valid inputs in this case). However, in this case, the full factorial experiment consists of only 16 unique cases (see Materials and Methods); consequently, eliminating the redundant rows in our gain matrix resulting in a reduced gain matrix with 15 input factors, some of which are multi-factor combinations (see Supplementary Information Table S4). Figure 4 shows a heat map of the elements of the gain matrix, indicating which input factor affects which glycan. The input factors include both main effects such as the concentration of media supplements (EDTA, MnCl_2_), times of addition (Mn T1, ED T1, etc.) as well as interaction effects, such as those between the times of addition (e.g., Mn T1-ED T1) or between concentration and the times of addition (e.g., EDTA—Mn T2, EDTA—Mn T1—ED T2, etc.). The colors indicate the direction of the effect (green for increase; red for decrease) and the intensity indicates magnitude. 

An examination of the heat map indicates that the glycan species FA2, FA2G1, and A2 are affected by most of the factors and their combinatorial interactions. For instance, the concentration of the most abundant glycoform, FA2 (which accounts for nearly 52% of the glycan concentration in the control sample), is affected by the concentration of MnCl_2_, the two-way interaction of MnCl_2_ and EDTA, and the late stage addition of MnCl_2_. In particular, a unit change in the concentration of MnCl_2_ causes an increase in the average concentration of FA2 (as indicated by the positive coefficient for the MnCl_2_ effect), while introducing MnCl_2_ on D3 causes a reduction in the average concentration of FA2. By contrast, the monogalactosylated form FA2G1 is not affected by changes in the media concentration of MnCl_2_; rather it is influenced by changes in the concentration of EDTA, the early addition of MnCl_2_, and the two-way interaction of MnCl_2_ and EDTA. Further, we observe that two of the interaction factors, Mn T1-ED T1 and Mn T2-ED T1, have statistically significant and opposing effects on the concentration of FA2G1. The factor Mn T1-ED T1 represents the two-way interaction of adding MnCl2 on D0 and adding EDTA on D0, whereas Mn T2-ED T1 represents the two-way interaction of adding MnCl2 on D3 when EDTA is introduced on D0. We observe therefore that the concentration of FA2G1 is affected by multiple input factors, including complex interactions between the amount of supplements added, and the times of addition of the supplements. One is thus able to assess the impact of each input factor on the response of all other individual glycan species in similar fashion. As indicated by the heat map, the effects of higher order interactions on most of the glycan species are negligible (if they exist at all) because estimates of the coefficients associated with most interaction effects are not statistically significant. 

## 4. Discussion

While the addition of specific media supplements has been studied widely for its effect on the quality attributes of mAbs, such studies have been limited to the introduction of media supplements exclusively at the start of the culture, and the results, when quantitative, have yielded only isolated single factor relationships. The results from the current study show that introducing media supplements at different time points during cell culture does in fact have an effect on cell growth conditions and antibody glycosylation distribution, and the effects can be quantified globally and potentially used to design effective control schemes. 

Specifically, we have shown that earlier addition of EDTA is detrimental to cell growth and results in a decrease in antibody titer. When EDTA was added on D0 (at the inoculation stage) the peak viable cell density (VCD) remained close to the seeding density, indicating a hampering of cell growth. This result is consistent with the well-known fact that EDTA is toxic to cells [35]. The decline in the viable cell densities due to EDTA addition during the growth phase can also be attributed to the removal of such trace metals as Ca^++^, Zn^++^ etc. that are essential for cell survival, from the media (as a result of EDTA’s chelating effect). By contrast, when both EDTA and MnCl_2_ were added on D0, the peak VCD increased two-fold. While this peak VCD is lower than the peak VCD of the control flask (5.78 × 10^6^ cells/mL), the increased viability can be attributed to the presence of excess MnCl_2_ titrating EDTA, resulting in reduced cytotoxicity. When EDTA was added on D3, the cells were in the middle of the growth phase and the addition of the cytotoxic EDTA hampered further growth, leading to a steep decline in the cell viability beyond D3 (Figure 1b). By contrast, when MnCl_2_ was added to the media (on D0 or D3) in the presence of EDTA, the cells did not experience a similar reduction in viability. When EDTA was added on D6, the cells were already at the end of the growth phase and hence the introduction of EDTA did not alter the cell viability. However, unlike the other cases where EDTA addition on D0 or D3 resulted in low titers, adding EDTA on D6 along with MnCl_2_ supplementation on D0, D3 or D6, resulted in end of run (EOR) titers higher than the titer value in the control flask. The increase in antibody titer in the presence of EDTA was observed by others as well [36,37] and is attributed to the inhibition of antibody reduction during cell lysis. Further, analyzing the EOR titer data using ANOVA shows that the factor coefficients for the concentration of EDTA, the concentration of MnCl_2_, and time of addition of EDTA are all statistically significant (at a significance level of α = 0.05). The expected change in the EOR titer in response to a unit change in any of these factors is quantified by the magnitude of that factor coefficient, while the sign of the factor coefficient indicates the direction of change. Thus, for example, a unit positive step change in the concentration of EDTA (with a factor coefficient of −0.017) or time of addition of EDTA (factor coefficient −0.032) results in a decrease in EOR titer; a unit positive step change in the concentration of MnCl_2_ (factor coefficient 0.008) causes an increase in titer. Quantifying the effect of these input factors on the EOR titer provides a rational basis for selecting which particular supplement to add, and how much of it to add, at a given stage of cell culture in order to maximize product yield. However, any media supplementation strategy must meet not just the desired specifications for final titer but also for product quality, i.e., to be effective, the implemented media supplementation strategy must not alter the glycan distributions significantly. 

The EOR titer represents the total amount of antibody accumulated at the end of the batch, while the measured glycan distribution represents the relative amount of each individual glycan isoform that has accumulated over the duration of the batch. Now, the relative amount of individual glycan species is a function of antibody productivity and it changes over the course of the batch. In our case, the addition of different media supplements at different stages of batch cultures affected both viability and antibody titer; consequently, the observed change in the glycan distribution has been induced by dynamic media supplementation and changes in productivity. To develop a mechanistic understanding of the effect of dynamic media supplementation on the glycosylation profile, therefore, we first decouple the effect of antibody productivity on the glycan distribution from the overall change observed at the end of the batch. One such decoupling approach involves estimating the mass fractions of specific glycoforms produced at different stages of cell culture using the expression [38]:

(4) $$f_{i}=\frac{\left[{{\mathrm{mAb}}_{i}|}_{t=\;t_{1}}\right]-\left[{{\mathrm{mAb}}_{i}|}_{t=\;t_{2}}\right]}{\left[{{\mathrm{mAb}}_{\mathrm{Tot}}|}_{t=\;t_{1}}\right]-\left[{{\mathrm{mAb}}_{Tot}|}_{t=\;t_{2}}\right]},$$

for *f_i_*, the fraction of mAb glycoform *i* produced in the time period [*t*_1_, *t*_2_] relative to the total amount of antibody secreted in the same period. However, we cannot use this expression for our purpose because we only measured EOR titer and final glycan distribution, not intermittent antibody titer or glycan concentration. Consequently, we propose an alternative metric-based solely on the final titer and glycan measurements. 

To illustrate, consider the glycan distribution in the control flask and in the flask with MnCl_2_ added on D6. In both flasks, the cell growth profile and antibody productivity will be the same until Day 6, when MnCl_2_ is introduced to the latter flask. Thus, the amount of *i*th glycoform fractions accumulated between Day 6 (D6) and the end of the run (EOR) for the two flasks can be written as:

(5) $$f_{\mathrm{Mn}6/i}=\frac{\left[{{\mathrm{mAb}}_{\mathrm{Mn}6/i}|}_{t=\;\mathrm{EOR}}\right]-\left[{{\mathrm{mAb}}_{\mathrm{Mn}6/i}|}_{t=\;D6}\right]}{\left[{{\mathrm{mAb}}_{\mathrm{Mn}6/Tot}|}_{t=\;\mathrm{EOR}}\right]-\left[{{\mathrm{mAb}}_{\mathrm{Mn}6/Tot}|}_{t=\;D6}\right]},$$

(6) $$f_{\mathrm{control}/i}=\frac{\left[{{\mathrm{mAb}}_{\mathrm{control}/i}|}_{t=\;\mathrm{EOR}}\right]-\left[{{\mathrm{mAb}}_{\mathrm{control}/i}|}_{t=\;D6}\right]}{\left[{{\mathrm{mAb}}_{\mathrm{control}/Tot}|}_{t=\;\mathrm{EOR}}\right]-\left[{{\mathrm{mAb}}_{\mathrm{control}/Tot}|}_{t=\;D6}\right]},$$

Recognizing that the D6 values in Equations (5) and (6) above are identical, we can eliminate the intermittent time point by simple arithmetic manipulations and obtain the change in the accumulation of the *i*th glycoform-based solely on EOR values, as:

(7) $$\Delta f_{i}=\frac{\left[{{\mathrm{mAb}}_{\mathrm{Mn}6/i}|}_{t=\;\mathrm{EOR}}\right]-\left[{{\mathrm{mAb}}_{\mathrm{control}/i}|}_{t=\;\mathrm{EOR}}\right]}{\left[{{\mathrm{mAb}}_{\mathrm{Mn}6/Tot}|}_{t=\;\mathrm{EOR}}\right]-\left[{{\mathrm{mAb}}_{\mathrm{control}/Tot}|}_{t=\;\mathrm{EOR}}\right]},$$

**Figure 5:**
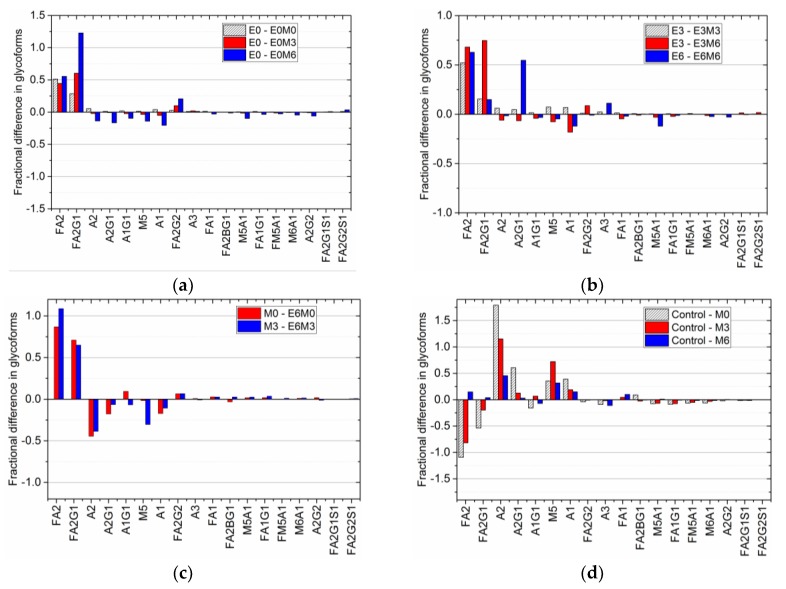
Fractional difference in glycoform distribution due to (**a**) MnCl_2_ addition on D0 (cross-hatched), D3 (red bar), and D6 (blue bar) after EDTA addition on D0, relative to the baseline glycan distribution in EDTA D0 supplemented flask; (**b**) MnCl_2_ addition on D3 (cross-hatched), and D6 (red) after EDTA addition on D3 relative to the baseline glycan distribution in EDTA D3 supplemented flask, and MnCl_2_ addition on D6 after EDTA addition on D6 (blue) relative to the baseline glycan distribution in EDTA D6 supplemented flask; (**c**) EDTA addition on D6 after MnCl_2_ addition on D0 (red) and D3 (blue) relative to the respective glycan distributions due to MnCl_2_ addition on D0 and D3; and (**d**) MnCl_2_ addition on D0 (cross-hatched), D3 (red bar), and D6 (blue bar) relative to the glycan distribution in the control case.

Thus, this fractional difference allows us to group together different experimental conditions with similar antibody titers, making it possible to compare final glycan distributions and hence quantify the effect of individual media supplements on the glycan profile appropriately. Such analyses extended to other experimental conditions produced the comparative fractional difference in the glycoform distribution shown in Figure 5. 

A comparison of the fractional difference in the glycan distribution in flasks where MnCl_2_ is added to D0 EDTA supplemented flasks relative to the glycan distribution in D0 EDTA supplemented flasks (Figure 5a), shows the following: an increase in the amount of FA2 (by nearly 50% in all cases), FA2G1 (by 29% during D0 supplementation, 60% during D3 supplementation, and 122% during D6 supplementation), and FA2G2 (by 3%, 10%, and 20% respectively), with a relative decrease in A2 and M5 by 2% and 4% when MnCl_2_ is added on D3, and nearly 14% when MnCl_2_ is added on D6. A similar trend is observed in the fractional difference in the glycan distribution in flasks where MnCl_2_ is added on D3 and D6 to D3 EDTA supplemented flasks, and when MnCl_2_ is added on D6 to D6 EDTA supplemented flasks (Figure 5b). Again, we notice an increase in FA2, FA2G1 and FA2G2, with a decrease in A2, M5, and A2G1 observed only when MnCl_2_ is added on day 6 after the addition of EDTA on D3. Previous studies have shown that adding MnCl_2_ produces an upregulation of galactosyltransferase enzymes [30], and subsequently in increased galactosylation [26,27]. Hence, the increase in the amount of FA2G1 and FA2G2 species can be attributed to the effect of late stage manganese addition on the galactosyltransferase enzyme. Figure 5c shows fractional difference when EDTA is added on D6 after MnCl_2_ has been added to the culture on D0 and D3. These differences are calculated relative to the glycan distribution observed due to the addition of MnCl_2_ on D3 and D6, respectively. We note that the fractional difference in the fucosylated species FA2 and FA2G1 is positive when EDTA is added after MnCl_2_ supplementation, indicating that the addition of EDTA increases the concentration of these species relative to their respective concentrations in MnCl_2_ supplemented cultures. Also, a comparison of the fractional difference in the glycan distribution when MnCl_2_ was introduced on D0, D3, or D6 relative to the glycan distribution in the control flask (Figure 5d), shows that the relative concentrations of FA2 and FA2G1 species decreased in flasks with D0, while late stage addition of MnCl_2_ did not have a significant effect on the overall glycan distribution. Taken together, our findings indicate that the latter addition of EDTA reverses the changes in the glycan distribution induced by MnCl_2_.

Fractional difference analysis helps to identify which glycan species are altered as a result of the addition of specific media supplements, but not why those particular glycan species changed. To identify the kinetic mechanisms underlying the changes observed in the glycan distribution upon adding MnCl_2_ to the media, we use an existing dynamic mathematical model of glycosylation [39] to simulate the system response under control conditions and when MnCl_2_ is added on D0, and obtain predictions of both the control and D0 glycan distributions (see Supplementary Information section S4 for details). The simulation results show that changes in the glycan distribution due to the addition of MnCl_2_ can be directly attributed to the changes in the reaction rates associated with the FucT enzyme.

In addition to fractional difference analysis, we use the glycan indices computed for each experimental condition (Table 2), as a metric for quantitative assessment of the change in the final glycosylation profile caused by the addition of different media supplements. Specifically, a comparison of the individual glycan indices for each condition against the corresponding values under control conditions allows us to establish, objectively, that altering the availability of MnCl_2_ in the media using a chelating agent reverses the changes in the glycan distribution. While MnCl_2_ addition on D0 resulted in a decrease in the fucosylation index from 79.9% in the control flask to 60.5%, we see that subsequent introduction of EDTA on either D0, D3, or D6 reversed that trend. Early stage addition of EDTA on D0 increased the fucosylation index to 82.46%, while adding EDTA on D3 and D6 resulted in fucosylation indices of 78.8%, and 76.5% respectively. Similarly, the decrease in the galactosylation index, upon adding MnCl_2_ on D0, from 17.9% in the control flask to 15.8%, could be reversed by subsequently adding EDTA on D3. Adding MnCl_2_ to the media on D3 reduced the fucosylation index to 61.9%, but if we then added EDTA on D6, the fucosylation index increased to 77.8%, which is comparable to the value of 79.9% in the control flask. It is important to note however, that the reversal in the glycan indices observed due to the addition of EDTA on D0 and D3 is achieved at the expense of reduced titer and reduced viability, as discussed above. The indication from our results is that changes in the glycan distribution due to MnCl_2_ addition can be reversed only when EDTA is added to the media after MnCl_2_ addition. Thus, the effect of MnCl_2_ supplementation can be reversed, without decreasing productivity, by adding EDTA on D6 to MnCl_2_ supplemented media, providing a means of ensuring higher productivity without altering glycan distribution.

Although such observations as these provide useful qualitative information about the system, they cannot be used to develop an automatic control strategy; that requires a quantitative representation (and hence understanding) of the effects of media supplementation on glycan distribution. Such quantitative representation can be obtained via formal analysis of the experimental data using analysis of variance (ANOVA) to generate the process gain matrix, **K**, as described in Materials and Methods. Singular value decomposition of the gain matrix **K** produces a (diagonal) matrix of singular values, **Σ**, and two unitary matrices, **W** and **V^T^**. Together these three matrices provide a particularly insightful representation of the process information encapsulated in the gain matrix, **K**: Equation (3) is transformed into a series of *n* individual and independent equations (see [29]) where, in each case, a linear combination of the original process input factors, with weighting coefficients from the matrix **W**, now constituting an “input mode”, **μ_i_**, is connected through the associated singular value **σ_i_**, to the corresponding linear combination of the output glycans, (with weighting coefficients from the matrix **V**), now constituting an output mode **η_i_** [29]. Furthermore, as a result of this decoupling transformation, the magnitude of each singular value naturally quantifies the extent to which the output mode in question will change in response to a change in the corresponding input mode. Thus, the larger the value of **σ_i_**, the greater will be the change in the corresponding output mode **η_i_** as a result of changes in the input mode **μ_i_**, so that output modes associated with larger values of **σ_i_** will be more “controllable” than modes associated with smaller values of **σ_i_**. 

The first ten singular values (**σ_1—_σ_10_**) for our experimental system are listed in Table 3 in decreasing order of magnitude. As modes associated with singular values of smaller magnitude are less controllable, we limit our analysis only to those modes that are practically controllable; we do this by using a threshold cutoff value, σ*, arbitrarily selected to be 0.50 in this example, thereby limiting our analysis to the first five singular values. From a process control perspective, modes associated with singular values below this threshold are considered to be of no practical importance since, for all intents and purposes, they are not controllable.

**Figure 6:**
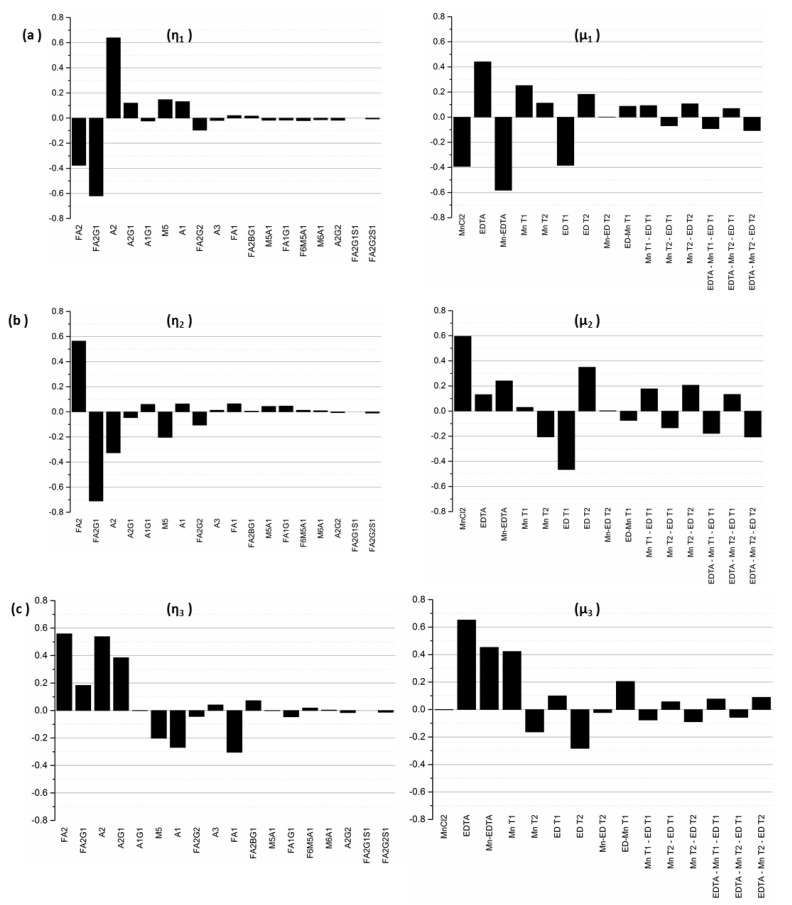

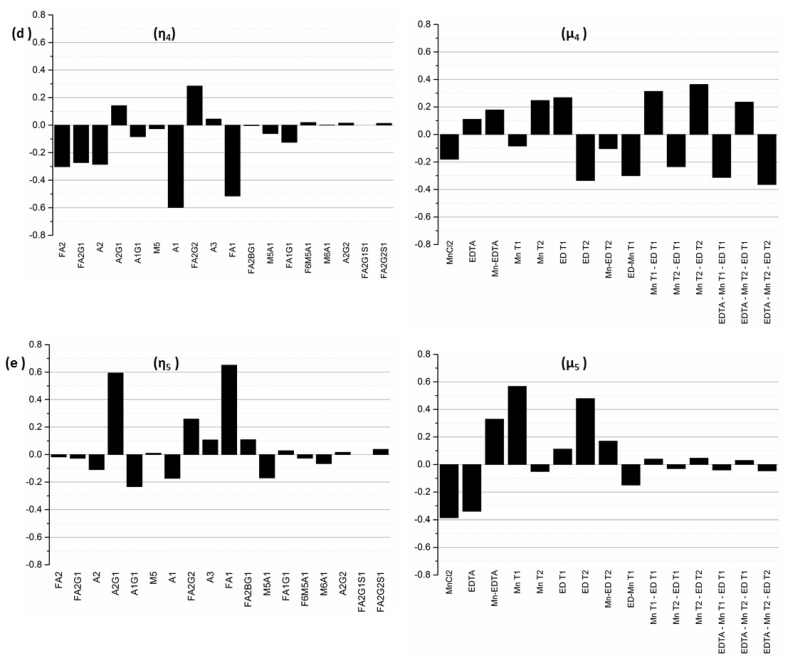
Graphical representation of the coefficients associated with the first five input and output modes (with σ_i_ ≥ σ* = 0.5) obtained from controllability analysis. (**a**) Output mode η_1_ and the corresponding input mode μ_1_; (**b**) Output mode η_2_ and the corresponding input mode μ_2_; (**c**) Output mode η_3_ and the corresponding input mode μ_3_; (**d**) Output mode η_4_ and the corresponding input mode μ_4_; (**e**) Output mode η_5_ and the corresponding input mode μ_5_.

Next, since by definition, each input–output mode pair represents the linear combination of output glycan species that can be controlled by manipulating the specific input factors in the corresponding input mode, the coefficients of each output factor in the output modes and of each input factor in the input modes provide further information about the relative influences exerted by each original input factor on each output factor. Specifically, the coefficient of a particular output factor in a particular mode represents the magnitude by which the relative percentages of those particular glycans will change in response to a unit change in the input mode. On the other hand, the coefficient of a particular input factor in the associated input mode corresponds to the relative contribution of that input factor to the unit change the input mode in question. Thus, the inputs with the largest coefficients in an input mode represent the dominant factors and hence the largest contributors to the influence of that mode, while the output glycans with the largest coefficients in an output mode represent those species whose relative percentages will change the most under the influence of the input modes. The input-output mode pairs and their associated coefficients are shown in Figure 6.

Because it is associated with the largest singular value of **σ_1_** = 6.62, the first output mode **η_1_** is the most controllable output mode. The value of σ_1_ represents the change in the overall output mode **η_1_** resulting from a unit change in the input mode **μ_1_**. For this output mode, we note that the dominant glycan species are A2, with a coefficient of 0.64, followed by FA2G1 (with a coefficient of −0.62), FA2 (−0.38), M5 (0.14), and A1 (0.13), indicating that a unit positive change in the input mode **μ_1_** will result in an increase in A2, M5, and A1, accompanied by a decrease in FA2G1 and FA2, each in the amount indicated by the identified coefficients. The biantennary species A2, with the largest coefficient, is the most controllable glycan in the first mode, followed by FA2G1 and FA2. The indicated coupling between the glycans A2, FA2G1, and FA2 makes sense because an increase in the afucosylated glycoforms occurs at the expense of the fucosylated forms, as our experimental results show. The associated input mode **μ_1_** is a linear combination of different input factors representing the media supplements MnCl_2_ and EDTA as well as the times of their addition. Mode **μ_1_** is primarily dominated by the interaction of MnCl2 and EDTA, and the concentrations of EDTA and MnCl_2_, with associated coefficients −0.58, 0.44, and −0.39 respectively, indicating that the addition of these two media supplements has opposing individual effects on output mode 1. Early stage addition of EDTA and MnCl_2_, denoted by the factors EDT1 and MnT1, with associated coefficients −0.38 and 0.25 respectively, also exert important influences on the first output mode. Based on the different elements that comprise the first input mode, this analysis indicates that one can control the glycans in output mode **η_1_** by adjusting the concentrations of the two supplements at the early stages of the cell culture.

The next controllable output mode **η_2_** is associated with the singular value **σ_2_** = 3.68, and the linear combination of glycans represented by this mode is dominated by the glycan species FA2G1, FA2, A2, M5, and FA2G2. The coefficients associated with these glycans are −0.71, 0.56, −0.33, −0.20 and −0.11, respectively, indicating that a unit positive change in the input mode **μ_2_** will cause a relative increase in FA2 while causing the other glycan species to decrease. The increase in FA2 coupled with the decrease in FA2G1 and FA2G2 indicates that perturbations to the input mode **μ_2_** affect the galactosylated species particularly. Since the singular values are arranged in decreasing order, the influence of mode **μ_2_** on output mode **η_2_** is less than that of **μ_1_** on mode **η_1_**. The largest coefficients in mode **μ_2_** are associated with the input factors MnCl_2_ (with a coefficient of 0.6), the early stage addition of EDTA denoted by factors EDT1 (with a coefficient of −0.47) and EDT2 (0.35), and the interaction effect of MnCl_2_ and EDTA (0.24). 

A unit positive change to the input mode **μ_3_** causes the following changes in the relative concentrations of the glycan species that comprise output mode **η_3_** (with **σ_3_** = 2.21): A2, A2G1, FA2, and FA2G1 increase, and M5, A1, and FA1 decrease, simultaneously. This indicates that input mode **μ_3_** can be used to increase the biantennary species, but at the expense of a (perfectly logical) concomitant decrease in the species that are upstream of these biantennary species. The input mode **μ_3_** is dominated by EDTA (with associated coefficient 0.65), the interaction effect of MnCl_2_ and EDTA (with coefficient 0.45), and the early stage addition of MnCl_2_ denoted by the factor MnT1). In each of the three input modes, the coefficients associated with the interaction effect of MnCl_2_ and EDTA indicate the importance of this combination input factor in altering the concentrations of the glycan species associated with the respective modes. 

The fourth and the fifth modes are less controllable, as they are associated with singular values of comparatively smaller magnitudes (**σ_4_** = 0.80 and **σ_5_** = 0.61). **η_4_** is dominated by glycans A1, A2, FA1, FA2, FA2G1, and FA2G2, while **η_5_** is dominated by FA1, A2G1, and FA2G2. The input mode **μ_4_** is dominated by the interaction effects Mn T2-ED T2 and EDTA*Mn T2-ED T2 representing the interaction between the time of addition of the media supplements. By contrast, the predominant factors in mode **μ_5_** are MnT1 and EDT2, which represent the addition of MnCl2 on D0 and EDTA on D3 respectively. 

It is worth mentioning the following facts about the controllability analysis presented here: (i) even though it is a theoretical analysis, the process gain matrix, **K** (on which the analysis is-based), was obtained entirely from experimental data. The information it contains about how changes in the inputs (amount of supplement added, and time of addition) affect glycan species, come directly from our designed experiments; (ii) Notwithstanding, a follow-up independent experimental validation of the modal analysis predictions (which contributor to which mode is affected in what manner) will be valuable. Such a validation experimental study, which lies outside the intended scope of this current work, is slated for future work. That study’s focus will not only be to validate the controllability analysis results; it will also encompass the more consequential design and implementation of a control system to achieve effective control of glycan distribution during a batch. 

For now, we note that the controllability analysis provides a data-based quantification of the effect that the addition of specified amounts of particular media supplements and the respective times of addition jointly have on the output glycan distribution at the end of the batch. As discussed above, introducing these media supplements dynamically also results in quantifiable changes in the antibody titer. Thus, the dynamic supplementation strategies discussed here present a challenging problem involving a trade-off between product yield and product quality. To be effective, a control strategy based on these considerations, must therefore be carefully designed to resolve these conflicts appropriately in order to optimize both the titer and the quality simultaneously. These matters will be addressed theoretically and experimentally in a follow up study.

In closing, we note that

(1) the relationships between time-dependent changes in media supplements and the corresponding changes in glycan distribution are (understandably) complex and not necessarily obvious or easily amenable to qualitative thinking; but
(2) controllability analysis via singular value decomposition, and the resulting input-output mode pairs determined specifically for our experimental system, using data from carefully designed experiments, have enabled us to identify which input factors are best manipulated, in order to effect changes in the relative percentage of specific glycan species;
(3) in addition, the coefficients in the equations representing the input and output modes allow us to quantify by how much we expect the glycan species to change in response to specific time-dependent media supplementation actions.

## 5. Conclusions

There is growing interest in evaluating the role of media supplements, especially MnCl_2_, in modulating glycoform distributions in mAbs. However, most media supplementation studies (where supplements are added to the media before starting the batch) do not take into consideration the impact of introducing media supplements at different stages of cell growth. In this paper, we have presented a systematic approach for evaluating the effects of time dependent media supplementation on the glycan profile, and provided a methodology for quantifying and analyzing the complex effects. Our results show that while it is important to consider which supplements are to be added to the media in order to alter specific glycan species, when they are to be added is just as important. In addition to this general observation about what to change and when, our results and analysis technique also demonstrate how to quantify “by how much” we can expect specific glycan species to change as a result of the changes in the media supplements. 

For instance, we observe that early stage addition of MnCl_2_ affects fucosylated species and alters the glycan distribution more significantly than a late stage addition. Similarly, early stage addition of EDTA affects not just the antibody titer, but also the relative percentages of biantennary and galactosylated species; late stage addition of EDTA does not alter the glycan distribution significantly. The glycan distribution profile is also affected by the addition of both EDTA and MnCl_2_ to the media at different time points and it is possible to develop a mechanistic understanding of the effect of individual media components on the glycan distribution by studying the fractional difference in the glycan distribution. However, we note that early stage addition of EDTA is detrimental to cell growth and antibody production, while late stage addition of EDTA improves titer with no appreciable effect on glycan distribution. This study does not recommend using EDTA as a media supplement—instead, our work presents a systematic approach to studying the complex interactions between media supplements when introduced at different time points during batch culture. Additionally, our results demonstrate that changes in the glycan distribution profile due to the addition of MnCl_2_ are not immutable; they can be reversed by adding EDTA after MnCl_2_ has been added to the media. We were able to identify the specific combinations of input factors which, when manipulated, result in quantifiable changes in the relative percentage of specific glycan species via controllability analysis using our experimental data. For the specific experimental system, our analyses show that A2, FA2G1 and FA2 are the most controllable glycan species whose concentrations can be changed by early stage supplementation of EDTA and late stage supplementation of MnCl_2_.

While this work has focused on establishing a rational framework for studying the influence of time-dependent media supplementation on the glycosylation profile, the techniques introduced here can be extended to tackle the complementary problem of designing and implementing appropriate control strategies to achieve desired glycan distribution profiles. Traditionally, the composition of cell culture media is fixed prior to starting the batch. However, we have demonstrated that introducing supplements at different points in time can influence both the productivity and the quality attribute of the antibody. Although we have examined only two specific media components in the current set of experiments, in principle, the systematic approach presented here can be extended easily to other media components such as amino acids, or trace metals. Future work will involve a comprehensive validation study where a control system will be designed on the basis of these results, and implemented on our experimental systems. 

## Figures and Tables

**Table 1:** Experimental conditions tested in the mixed level factorial design.

Experimental Condition	MnCl_2_ Conc. (mM)	EDTA Conc. (mM)	Time of Addition of MnCl_2_^1^	Time of Addition of EDTA ^1^	Label
1	0.01	0	D0	D0	Control
2	0.01	0.08	D0	D0	ED D0
3	0.04	0.08	D0	D0	Mn D0/ED D0
4	0.04	0.08	D3	D0	Mn D3/ED D0
5	0.04	0.08	D6	D0	Mn D6/ED D0
6	0.01	0.08	D0	D3	ED D3
7	0.04	0.08	D0	D3	Mn D0/ED D3
8	0.04	0.08	D3	D3	Mn D3/ED D3
9	0.04	0.08	D6	D3	Mn D6/ED D3
10	0.01	0.08	D0	D6	ED D6
11	0.04	0.08	D0	D6	Mn D0/ED D6
12	0.04	0.08	D3	D6	Mn D3/ED D6
13	0.04	0.08	D6	D6	Mn D6/ED D6
14	0.04	0	D0	D0	Mn D0
15	0.04	0	D3	D0	Mn D3
16	0.04	0	D6	D0	Mn D6

^1^ D0, D3 and D6 refer to Day 0, Day 3 and Day 6 after inoculation, respectively.

**Table 2:** Galactosylation index (GI) and fucosylation index (FI) for each experimental condition.

Experimental Condition ^1^	Galactosylation Index (GI)	Fucosylation Index (FI)
Control	17.9	79.9
Mn D0	15.8	60.5
Mn D0/ED D0	20.6	82.5
Mn D0/ED D3	18.0	78.8
Mn D0/ED D6	19.3	76.5
Mn D3	15.7	61.8
Mn D3/ED D0	25.2	88.6
Mn D3/ED D3	15.1	73.9
Mn D3/ED D6	18.0	77.8
Mn D6	16.7	76.3
Mn D6/ED D0	24.8	90.3
Mn D6/ED D3	19.9	86.4
Mn D6/ED D6	17.9	77.3
ED D0	21.6	81.0
ED D3	16.5	75.3
ED D6	16.9	77.7

^1^ Mn and ED refer to the media supplements MnCl_2_ and EDTA, while D0, D3 and D6 refer to Day 0, Day 3 and Day 6 after inoculation, respectively.

**Table 3:** Singular values obtained from singular value decomposition of the matrix of significant factor coefficients.

σ_1_	6.22
σ_2_	3.68
σ_3_	2.21
σ_4_	0.80
σ_5_	0.61
σ_6_	0.45
σ_7_	0.41
σ_8_	0.31
σ_9_	0.10
σ_10_	0.05

## Supplementary Materials

The following are available online at http://www.mdpi.com/2073-4468/7/1/1/s1, Table S1: Experimentally observed glycan species and their masses; Table S2: Full factorial experimental design table; Table S3: Gain matrix generated from statistically significant (*p* ≤ 0.05) coefficients obtained from ANOVA of the full factorial design experimental data; Table S4: Main and interaction effects and corresponding confounding factors; Table S5: “Reduced” gain matrix (**K**) obtained by eliminating the redundant rows from the gain matrix listed in Table S3; Table S6: Unitary matrix **W** obtained from SVD of gain matrix **K**; Table S7: Diagonal matrix of singular values (**Σ**) obtained from SVD of reduced gain matrix **K**; Table S8: Unitary matrix **V^T^** obtained from SVD of reduced gain matrix **K**; Table S9: Reaction rules for generating the glycan reaction network used in simulations of the dynamic glycosylation model; Table S10: Enzyme and co-substrate concentrations used in the simulations of the dynamic glycosylation model; Figure S1: Comparison of experimental data and model fit for the glycan distribution profile obtained from (a) control flask; and (b) when MnCl_2_ is added on D0; Figure S2: Normalized glucose concentration data for each condition tested; Figure S3: Average relative glycan percentage of IgG1 glycans produced in CHO-K1 cells.

Supplementary File 1
