# Supplementary material for: Controlling the Glycosylation Profile in mAbs Using Time-Dependent Media Supplementation
Source: Antibodies (Basel). 2017 Dec 21;7(1):1. doi: 10.3390/antib7010001 (PMC6698858; doi:10.3390/antib7010001)
Supplement: Supplementary File 1 [file antibodies-07-00001-s001.doc]

**Controlling the glycosylation profile in mAbs using time-dependent media supplementation**

Devesh Radhakrishnan^1^, Anne S. Robinson^1, 2^, Babatunde A. Ogunnaike^1^

^1^ Department of Chemical and Biomolecular Engineering, University of Delaware, Newark, Delaware 19716, USA

^2^ Department of Chemical and Biomolecular Engineering, Tulane University, New Orleans, Louisiana 70118, USA

**Corresponding Author:**

Babatunde A. Ogunnaike,

University of Delaware,

Newark, DE 19716

302-831-4504

[ogunnaike@udel.edu](mailto:ogunnaike@udel.edu)

302-831-1048 (fax)

**Supplementary Information S1**

Table S1: Experimentally observed glycan species and their masses. Glycan structures drawn using GlycoForm software (McDonald et al. 2010), with sugar symbol set specified as per Consortium of Functional Glycomics.

| Glycan species | Structure | Mass (m/z) |
| --- | --- | --- |
| FA2 | 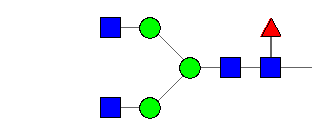 | 1835.92 |
| FA2G1 | 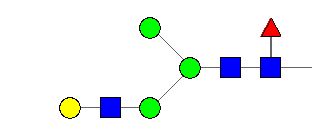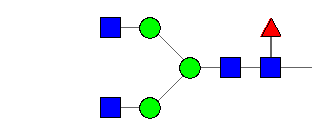 | 2040.02 |
| A2 | 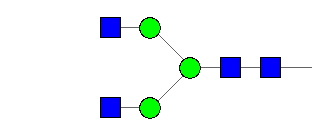 | 1661.83 |
| A2G1 | 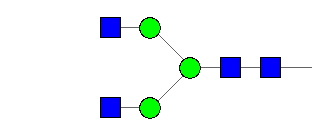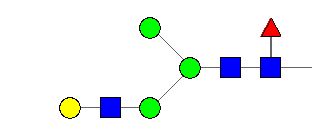 | 1865.93 |
| A1G1 | 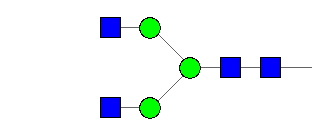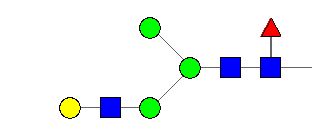 | 1620.8 |
| M5 | 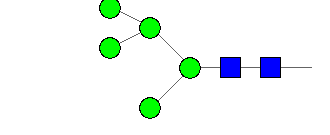 | 1579.78 |
| A1 | 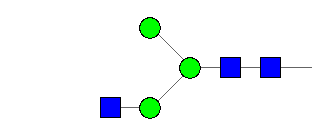 | 1416.7 |
| FA2G2 | 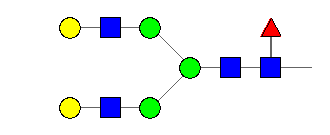 | 2244.12 |
| A3 | 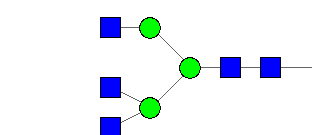 | 1906.9 |
| FA1 | 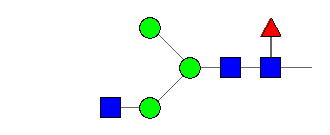 | 1590.79 |
| FA2BG1 | 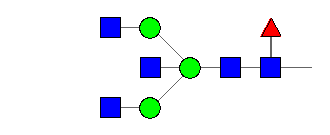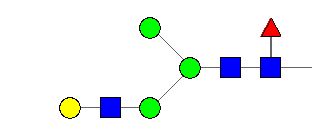 | 2285.2 |
| M5A1 | 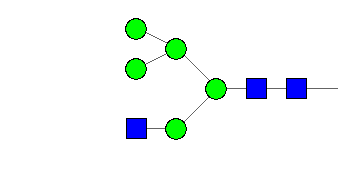 | 1824.9 |
| FA1G1 | 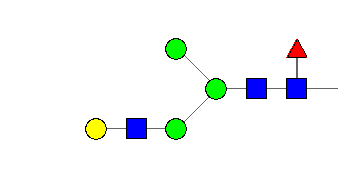 | 1794.89 |
| FM5A1 | 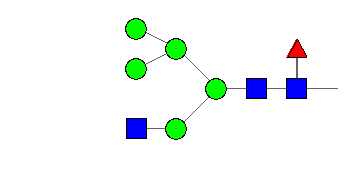 | 1998.9 |
| M6A1 | 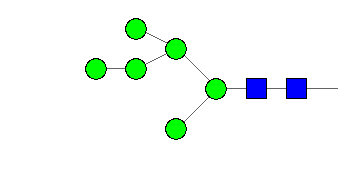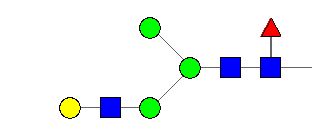 | 2029.0 |
| A2G2 | 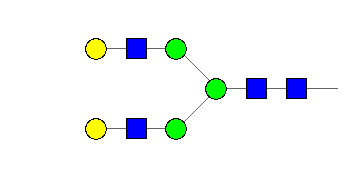 | 2070.036 |
| FA2G1S1 | 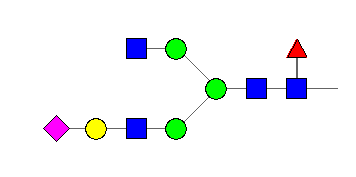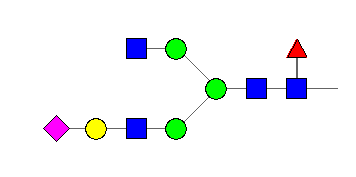 | 2401.19 |
| FA2G2S1 | 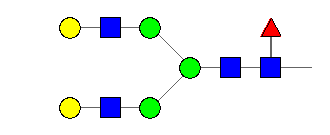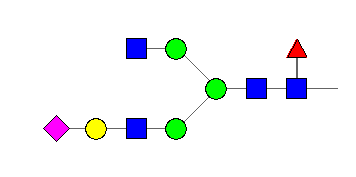 | 2605.29 |

**Supplementary Information S2**

**Full factorial experimental design table:** The accompanying table lists the full factorial (2^2^,3^2^) experimental design as well as the labels associated with each experimental condition. The 36 experimental conditions listed here reduce to a set of 16 unique experiments.

Table S2: Full factorial experimental design table

| **Experiment** | **MnCl_2_ level** | **EDTA level** | **MnCl_2_ Addition** | **EDTA Addition** | **Label** |
| --- | --- | --- | --- | --- | --- |
| 1 | -1 | -1 | -1 | -1 | Control |
| 2 | -1 | -1 | -1 | 0 |
| 3 | -1 | -1 | -1 | +1 |
| 4 | -1 | -1 | 0 | -1 |
| 5 | -1 | -1 | 0 | 0 |
| 6 | -1 | -1 | 0 | +1 |
| 7 | -1 | -1 | +1 | -1 |
| 8 | -1 | -1 | +1 | 0 |
| 9 | -1 | -1 | +1 | +1 |
| 10 | -1 | +1 | -1 | -1 | ED D0 |
| 11 | -1 | +1 | 0 | -1 |
| 12 | -1 | +1 | +1 | -1 |
| 13 | -1 | +1 | -1 | 0 | ED D3 |
| 14 | -1 | +1 | 0 | 0 |
| 15 | -1 | +1 | +1 | 0 |
| 16 | -1 | +1 | -1 | +1 | ED D6 |
| 17 | -1 | +1 | 0 | +1 |
| 18 | -1 | +1 | +1 | +1 |
| 19 | +1 | -1 | -1 | -1 | Mn D0 |
| 20 | +1 | -1 | -1 | 0 |
| 21 | +1 | -1 | -1 | +1 |
| 22 | +1 | -1 | 0 | -1 | Mn D3 |
| 23 | +1 | -1 | 0 | 0 |
| 24 | +1 | -1 | 0 | +1 |
| 25 | +1 | -1 | +1 | -1 | Mn D6 |
| 26 | +1 | -1 | +1 | 0 |
| 27 | +1 | -1 | +1 | +1 |
| 28 | +1 | +1 | -1 | -1 | ED D0/ Mn D0 |
| 29 | +1 | +1 | -1 | 0 | ED D3/ Mn D0 |
| 30 | +1 | +1 | -1 | +1 | ED D6/ Mn D0 |
| 31 | +1 | +1 | 0 | -1 | ED D0/Mn D3 |
| 32 | +1 | +1 | 0 | 0 | ED D3/ Mn D3 |
| 33 | +1 | +1 | 0 | +1 | ED D6/ Mn D3 |
| 34 | +1 | +1 | +1 | -1 | ED D0/Mn D6 |
| 35 | +1 | +1 | +1 | 0 | ED D3/Mn D6 |
| 36 | +1 | +1 | +1 | +1 | ED D6/Mn D6 |

†D0, D3, D6 refer to the time of addition of the supplement MnCl_2_ (Mn) or EDTA (ED) on day 0, day 3, or day 6 respectively.

**Supplementary Information S3**

The experiments in this study were carried out according to a 2^2^3^2^ mixed factorial design. A full factorial design would have involved 36 experiments, many of which are not unique. Hence, we reduced these 36 experiments to the set of 16 unique experiments that were performed. Table S3 shows the estimates of the factor coefficients obtained by analyzing the factorial design data. We select the statistically significant factor coefficients (at the significance level of α = 0.05), and set all other coefficients to zero. The alias structure (confounding factors) for this design is listed in Table S4 and the corresponding “reduced” gain matrix **K** obtained by eliminating the aliased factor coefficients is listed in Table S5. Tables S6-S8 show the results of the singular value decomposition (SVD) of **K,** i.e., the diagonal matrix **Σ** of the singular values of **K**, and the corresponding unitary matrices **W** and **V^T^**.

Table S3: Gain matrix generated from statistically significant (p ≤0.05) coefficients obtained from ANOVA of the full factorial design experimental data

|  | **FA2** | **FA2G1** | **A2** | **A2G1** | **A1G1** | **M5** | **A1** | **FA2G2** | **A3** | **FA1** | **FA2BG1** | **M5A1** | **FA1G1** | **FM5A1** | **M6A1** | **A2G2** | **FA2G1S1** | **FA2G2S1** |
| --- | --- | --- | --- | --- | --- | --- | --- | --- | --- | --- | --- | --- | --- | --- | --- | --- | --- | --- |
| **MnCl2** | 2.17 | 0.00 | -2.21 | -0.50 | 0.37 | -0.86 | 0.00 | 0.00 | 0.00 | 0.00 | 0.00 | 0.27 | 0.22 | 0.13 | 0.13 | 0.07 | 0.00 | 0.00 |
| **EDTA** | 0.00 | -1.79 | 2.34 | 0.80 | 0.00 | 0.00 | 0.00 | -0.48 | 0.00 | -0.55 | 0.12 | 0.00 | -0.09 | 0.00 | 0.00 | -0.07 | 0.00 | -0.05 |
| **Mn Time 1** | 0.00 | -0.87 | 1.45 | 0.78 | 0.00 | 0.00 | 0.00 | 0.00 | 0.00 | 0.00 | 0.19 | 0.00 | 0.00 | 0.00 | 0.00 | 0.00 | 0.00 | 0.00 |
| **Mn Time 2** | -1.08 | 0.00 | 0.45 | 0.00 | 0.00 | 0.00 | 0.00 | 0.00 | 0.00 | 0.00 | 0.00 | 0.00 | 0.00 | 0.00 | 0.00 | 0.00 | 0.00 | 0.00 |
| **ED Time 1** | 0.00 | 2.67 | -0.92 | 0.00 | 0.00 | 0.00 | -0.57 | 0.59 | 0.00 | -0.32 | 0.00 | 0.00 | 0.00 | 0.10 | 0.09 | 0.12 | 0.00 | 0.06 |
| **ED Time 2** | 0.00 | -1.66 | 0.00 | 0.00 | 0.00 | 0.00 | 0.51 | -0.27 | 0.00 | 0.62 | 0.00 | 0.00 | 0.15 | 0.00 | 0.00 | 0.00 | 0.00 | 0.00 |
| **MnCl2 – EDTA** | 2.35 | 1.76 | -2.12 | 0.00 | 0.00 | -0.99 | -0.87 | 0.18 | 0.20 | -0.23 | 0.00 | 0.00 | 0.00 | 0.07 | 0.00 | 0.00 | 0.00 | 0.00 |
| **MnCl2 - Mn Time1** | 0.00 | 0.87 | -1.45 | -0.78 | 0.00 | 0.00 | 0.00 | 0.00 | 0.00 | 0.00 | -0.19 | 0.00 | 0.00 | 0.00 | 0.00 | 0.00 | 0.00 | 0.00 |
| **MnCl2 - Mn Time2** | 1.08 | 0.00 | -0.45 | 0.00 | 0.00 | 0.00 | 0.00 | 0.00 | 0.00 | 0.00 | 0.00 | 0.00 | 0.00 | 0.00 | 0.00 | 0.00 | 0.00 | 0.00 |
| **MnCl2 - ED Time1** | 0.00 | 0.00 | 0.00 | 0.00 | 0.00 | 0.00 | 0.00 | 0.00 | 0.00 | 0.00 | 0.00 | 0.00 | 0.00 | 0.00 | 0.00 | 0.00 | 0.00 | 0.00 |
| **MnCl2 - ED Time2** | 0.00 | 0.00 | 0.00 | 0.00 | 0.00 | 0.00 | 0.00 | 0.00 | 0.00 | 0.16 | 0.00 | 0.00 | 0.00 | 0.00 | 0.00 | 0.00 | 0.00 | 0.00 |
| **EDTA - Mn Time 1** | 0.00 | 0.00 | 0.84 | 0.00 | 0.00 | 0.00 | 0.00 | 0.00 | 0.00 | 0.00 | 0.00 | 0.00 | 0.00 | 0.00 | 0.00 | 0.00 | 0.00 | 0.00 |
| **EDTA - Mn Time 2** | 0.00 | 0.00 | 0.00 | 0.00 | 0.00 | 0.00 | 0.00 | 0.00 | 0.00 | 0.00 | 0.00 | 0.00 | 0.00 | 0.00 | 0.00 | 0.00 | 0.00 | 0.00 |
| **EDTA - ED Time 1** | 0.00 | -2.67 | 0.92 | 0.00 | 0.00 | 0.00 | 0.57 | -0.59 | 0.00 | 0.32 | 0.00 | 0.00 | 0.00 | -0.10 | -0.09 | -0.12 | 0.00 | -0.06 |
| **EDTA - ED Time 2** | 0.00 | 1.66 | 0.00 | 0.00 | 0.00 | 0.00 | -0.51 | 0.27 | 0.00 | -0.62 | 0.00 | 0.00 | -0.15 | 0.00 | 0.00 | 0.00 | 0.00 | 0.00 |
| **Mn T1 - ED T1** | 0.00 | -0.92 | 0.00 | 0.00 | 0.00 | 0.00 | 0.00 | 0.00 | 0.00 | 0.00 | 0.00 | 0.00 | 0.00 | 0.00 | 0.00 | 0.00 | 0.00 | 0.00 |
| **Mn T1 - ED T2** | 0.00 | 0.00 | 0.00 | 0.00 | 0.00 | 0.00 | 0.00 | 0.00 | 0.00 | 0.00 | 0.00 | 0.00 | 0.00 | 0.00 | 0.00 | 0.00 | 0.00 | 0.00 |
| **Mn T2 - ED T1** | 0.00 | 0.69 | 0.00 | 0.00 | 0.00 | 0.00 | 0.00 | 0.00 | 0.00 | 0.00 | 0.00 | 0.00 | 0.00 | 0.00 | 0.00 | 0.00 | 0.00 | 0.00 |
| **Mn T2 - ED T2** | 0.00 | -1.07 | 0.00 | 0.00 | 0.00 | 0.00 | 0.00 | 0.00 | 0.00 | 0.00 | 0.00 | 0.00 | 0.00 | 0.00 | 0.00 | 0.00 | 0.00 | 0.00 |
| **MnCl2-EDTA-Mn T1** | 0.00 | 0.00 | -0.84 | 0.00 | 0.00 | 0.00 | 0.00 | 0.00 | 0.00 | 0.00 | 0.00 | 0.00 | 0.00 | 0.00 | 0.00 | 0.00 | 0.00 | 0.00 |
| **MnCl2-EDTA-Mn T2** | 0.00 | 0.00 | 0.00 | 0.00 | 0.00 | 0.00 | 0.00 | 0.00 | 0.00 | 0.00 | 0.00 | 0.00 | 0.00 | 0.00 | 0.00 | 0.00 | 0.00 | 0.00 |
| **MnCl2-EDTA-ED T1** | 0.00 | 0.00 | 0.00 | 0.00 | 0.00 | 0.00 | 0.00 | 0.00 | 0.00 | 0.00 | 0.00 | 0.00 | 0.00 | 0.00 | 0.00 | 0.00 | 0.00 | 0.00 |
| **MnCl2-EDTA-ED T2** | 0.00 | 0.00 | 0.00 | 0.00 | 0.00 | 0.00 | 0.00 | 0.00 | 0.00 | -0.16 | 0.00 | 0.00 | 0.00 | 0.00 | 0.00 | 0.00 | 0.00 | 0.00 |
| **MnCl2 - Mn T1 - ED T1** | 0.00 | 0.92 | 0.00 | 0.00 | 0.00 | 0.00 | 0.00 | 0.00 | 0.00 | 0.00 | 0.00 | 0.00 | 0.00 | 0.00 | 0.00 | 0.00 | 0.00 | 0.00 |
| **MnCl2 - Mn T1 - ED T2** | 0.00 | 0.00 | 0.00 | 0.00 | 0.00 | 0.00 | 0.00 | 0.00 | 0.00 | 0.00 | 0.00 | 0.00 | 0.00 | 0.00 | 0.00 | 0.00 | 0.00 | 0.00 |
| **MnCl2 - Mn T2 - ED T1** | 0.00 | -0.69 | 0.00 | 0.00 | 0.00 | 0.00 | 0.00 | 0.00 | 0.00 | 0.00 | 0.00 | 0.00 | 0.00 | 0.00 | 0.00 | 0.00 | 0.00 | 0.00 |
| **MnCl2 - Mn T2 - ED T2** | 0.00 | 1.07 | 0.00 | 0.00 | 0.00 | 0.00 | 0.00 | 0.00 | 0.00 | 0.00 | 0.00 | 0.00 | 0.00 | 0.00 | 0.00 | 0.00 | 0.00 | 0.00 |
| **EDTA - Mn T1 - ED T1** | 0.00 | 0.92 | 0.00 | 0.00 | 0.00 | 0.00 | 0.00 | 0.00 | 0.00 | 0.00 | 0.00 | 0.00 | 0.00 | 0.00 | 0.00 | 0.00 | 0.00 | 0.00 |
| **EDTA - Mn T1 - ED T2** | 0.00 | 0.00 | 0.00 | 0.00 | 0.00 | 0.00 | 0.00 | 0.00 | 0.00 | 0.00 | 0.00 | 0.00 | 0.00 | 0.00 | 0.00 | 0.00 | 0.00 | 0.00 |
| **EDTA - Mn T2 - ED T1** | 0.00 | -0.69 | 0.00 | 0.00 | 0.00 | 0.00 | 0.00 | 0.00 | 0.00 | 0.00 | 0.00 | 0.00 | 0.00 | 0.00 | 0.00 | 0.00 | 0.00 | 0.00 |
| **EDTA - Mn T2 - ED T2** | 0.00 | 1.07 | 0.00 | 0.00 | 0.00 | 0.00 | 0.00 | 0.00 | 0.00 | 0.00 | 0.00 | 0.00 | 0.00 | 0.00 | 0.00 | 0.00 | 0.00 | 0.00 |
| **MnCl2 - EDTA - Mn T1 - ED T1** | 0.00 | -0.92 | 0.00 | 0.00 | 0.00 | 0.00 | 0.00 | 0.00 | 0.00 | 0.00 | 0.00 | 0.00 | 0.00 | 0.00 | 0.00 | 0.00 | 0.00 | 0.00 |
| **MnCl2 - EDTA - Mn T1 - ED T2** | 0.00 | 0.00 | 0.00 | 0.00 | 0.00 | 0.00 | 0.00 | 0.00 | 0.00 | 0.00 | 0.00 | 0.00 | 0.00 | 0.00 | 0.00 | 0.00 | 0.00 | 0.00 |
| **MnCl2 - EDTA - Mn T2 - ED T1** | 0.00 | 0.69 | 0.00 | 0.00 | 0.00 | 0.00 | 0.00 | 0.00 | 0.00 | 0.00 | 0.00 | 0.00 | 0.00 | 0.00 | 0.00 | 0.00 | 0.00 | 0.00 |
| **MnCl2 - EDTA - Mn T2 - ED T2** | 0.00 | -1.07 | 0.00 | 0.00 | 0.00 | 0.00 | 0.00 | 0.00 | 0.00 | 0.00 | 0.00 | 0.00 | 0.00 | 0.00 | 0.00 | 0.00 | 0.00 | 0.00 |

Table S4: Main and interaction effects and corresponding confounding factors

| EDTA |  |  |  |
| --- | --- | --- | --- |
| MnCl_2_ |  |  |  |
| MnCl_2_ – EDTA |  |  |  |
| ED T1 | = | -1*EDTA - ED T1 | |
| ED T2 | = | -1*EDTA - ED T2 | |
| Mn T1 | = | -1*MnCl_2_ - Mn T1 | |
| Mn T2 | = | -1*MnCl_2_ - Mn T2 | |
| Mn T1 - ED T1 | = | -1*MnCl_2_ - Mn T1 - ED T1 | |
| Mn T2 - ED T1 | = | -1*MnCl_2_ - Mn T2 - ED T1 | |
| Mn T2 - ED T2 | = | -1*MnCl_2_ - Mn T2 - ED T2 | |
| Mn T1 - ED T2 | = | -1*MnCl_2_ - Mn T1 - ED T2 | |
| EDTA - Mn T1 | = | -1*MnCl_2_-EDTA-Mn T1 | |
| MnCl_2_ - ED T2 | = | -1*MnCl_2_-EDTA-ED T2 | |
| EDTA - Mn T2 | = | -1*MnCl_2_-EDTA-Mn T2 | |
| EDTA - Mn T1 - ED T1 | = | -1*MnCl_2_ - EDTA - Mn T1 - ED T1 | |
| EDTA - Mn T2 - ED T1 | = | -1*MnCl_2_ - EDTA - Mn T2 - ED T1 | |
| EDTA - Mn T2 - ED T2 | = | -1*MnCl_2_ - EDTA - Mn T2 - ED T2 | |
| EDTA - Mn T1 - ED T2 | = | -1*MnCl_2_ - EDTA - Mn T1 - ED T2 | |
| MnCl_2_ - ED T1 | = | -1*MnCl_2_-EDTA-ED T1 | |

Table S5: “Reduced” gain matrix (**K**) obtained by eliminating the redundant rows from the gain matrix listed in Table S3

|  | **FA2** | **FA2G1** | **A2** | **A2G1** | **A1G1** | **M5** | **A1** | **FA2G2** | **A3** | **FA1** | **FA2BG1** | **M5A1** | **FA1G1** | **FM5A1** | **M6A1** | **A2G2** | **FA2G1S1** | **FA2G2S1** |
| --- | --- | --- | --- | --- | --- | --- | --- | --- | --- | --- | --- | --- | --- | --- | --- | --- | --- | --- |
| **MnCl_2_** | 2.17 | 0.00 | -2.21 | -0.50 | 0.37 | -0.86 | 0.00 | 0.00 | 0.00 | 0.00 | 0.00 | 0.27 | 0.22 | 0.13 | 0.13 | 0.07 | 0.00 | 0.00 |
| **EDTA** | 0.00 | -1.79 | 2.34 | 0.80 | 0.00 | 0.00 | 0.00 | -0.48 | 0.00 | -0.55 | 0.12 | 0.00 | -0.09 | 0.00 | 0.00 | -0.07 | 0.00 | -0.05 |
| **MnCl_2_ – EDTA** | 2.35 | 1.76 | -2.12 | 0.00 | 0.00 | -0.99 | -0.87 | 0.18 | 0.20 | -0.23 | 0.00 | 0.00 | 0.00 | 0.07 | 0.00 | 0.00 | 0.00 | 0.00 |
| **Mn Time 1** | 0.00 | -0.87 | 1.45 | 0.78 | 0.00 | 0.00 | 0.00 | 0.00 | 0.00 | 0.00 | 0.19 | 0.00 | 0.00 | 0.00 | 0.00 | 0.00 | 0.00 | 0.00 |
| **Mn Time 2** | -1.08 | 0.00 | 0.45 | 0.00 | 0.00 | 0.00 | 0.00 | 0.00 | 0.00 | 0.00 | 0.00 | 0.00 | 0.00 | 0.00 | 0.00 | 0.00 | 0.00 | 0.00 |
| **ED Time 1** | 0.00 | 2.67 | -0.92 | 0.00 | 0.00 | 0.00 | -0.57 | 0.59 | 0.00 | -0.32 | 0.00 | 0.00 | 0.00 | 0.10 | 0.09 | 0.12 | 0.00 | 0.06 |
| **ED Time 2** | 0.00 | -1.66 | 0.00 | 0.00 | 0.00 | 0.00 | 0.51 | -0.27 | 0.00 | 0.62 | 0.00 | 0.00 | 0.15 | 0.00 | 0.00 | 0.00 | 0.00 | 0.00 |
| **MnCl_2_ - ED Time2** | 0.00 | 0.00 | 0.00 | 0.00 | 0.00 | 0.00 | 0.00 | 0.00 | 0.00 | 0.16 | 0.00 | 0.00 | 0.00 | 0.00 | 0.00 | 0.00 | 0.00 | 0.00 |
| **EDTA - Mn Time 1** | 0.00 | 0.00 | 0.84 | 0.00 | 0.00 | 0.00 | 0.00 | 0.00 | 0.00 | 0.00 | 0.00 | 0.00 | 0.00 | 0.00 | 0.00 | 0.00 | 0.00 | 0.00 |
| **Mn T1 - ED T1** | 0.00 | -0.92 | 0.00 | 0.00 | 0.00 | 0.00 | 0.00 | 0.00 | 0.00 | 0.00 | 0.00 | 0.00 | 0.00 | 0.00 | 0.00 | 0.00 | 0.00 | 0.00 |
| **Mn T2 - ED T1** | 0.00 | 0.69 | 0.00 | 0.00 | 0.00 | 0.00 | 0.00 | 0.00 | 0.00 | 0.00 | 0.00 | 0.00 | 0.00 | 0.00 | 0.00 | 0.00 | 0.00 | 0.00 |
| **Mn T2 - ED T2** | 0.00 | -1.07 | 0.00 | 0.00 | 0.00 | 0.00 | 0.00 | 0.00 | 0.00 | 0.00 | 0.00 | 0.00 | 0.00 | 0.00 | 0.00 | 0.00 | 0.00 | 0.00 |
| **EDTA - Mn T1 - ED T1** | 0.00 | 0.92 | 0.00 | 0.00 | 0.00 | 0.00 | 0.00 | 0.00 | 0.00 | 0.00 | 0.00 | 0.00 | 0.00 | 0.00 | 0.00 | 0.00 | 0.00 | 0.00 |
| **EDTA - Mn T2 - ED T1** | 0.00 | -0.69 | 0.00 | 0.00 | 0.00 | 0.00 | 0.00 | 0.00 | 0.00 | 0.00 | 0.00 | 0.00 | 0.00 | 0.00 | 0.00 | 0.00 | 0.00 | 0.00 |
| **EDTA - Mn T2 - ED T2** | 0.00 | 1.07 | 0.00 | 0.00 | 0.00 | 0.00 | 0.00 | 0.00 | 0.00 | 0.00 | 0.00 | 0.00 | 0.00 | 0.00 | 0.00 | 0.00 | 0.00 | 0.00 |

Table S6: Unitary matrix **W** obtained from SVD of gain matrix **K**

|  | **η1** | **η2** | **η3** | **η4** | **η5** | **η6** | **η7** | **η8** | **η9** | **η10** | **η11** | **η12** | **η13** | **η14** | **η15** | **η16** | **η17** | **η18** |
| --- | --- | --- | --- | --- | --- | --- | --- | --- | --- | --- | --- | --- | --- | --- | --- | --- | --- | --- |
| **FA2** | -0.38 | 0.56 | 0.56 | -0.30 | -0.02 | 0.04 | 0.35 | -0.10 | 0.01 | 0.00 | 0.00 | 0.00 | 0.00 | 0.00 | 0.00 | 0.00 | 0.00 | 0.00 |
| **FA2G1** | -0.62 | -0.71 | 0.18 | -0.27 | -0.03 | -0.03 | -0.04 | 0.04 | 0.00 | 0.00 | 0.00 | 0.00 | 0.00 | 0.00 | 0.00 | 0.00 | 0.00 | 0.00 |
| **A2** | 0.64 | -0.33 | 0.54 | -0.29 | -0.11 | 0.00 | 0.01 | -0.32 | 0.03 | 0.01 | 0.00 | 0.00 | 0.00 | 0.00 | 0.00 | 0.00 | 0.00 | 0.00 |
| **A2G1** | 0.12 | -0.05 | 0.39 | 0.14 | 0.59 | 0.19 | -0.11 | 0.60 | -0.02 | -0.01 | 0.02 | -0.06 | 0.02 | -0.19 | -0.06 | -0.07 | 0.00 | -0.03 |
| **A1G1** | -0.02 | 0.06 | 0.00 | -0.08 | -0.23 | 0.40 | -0.23 | 0.01 | -0.19 | -0.44 | 0.39 | 0.44 | 0.25 | -0.25 | -0.05 | 0.01 | 0.00 | -0.10 |
| **M5** | 0.15 | -0.20 | -0.20 | -0.03 | 0.01 | 0.14 | 0.82 | 0.22 | 0.13 | -0.25 | -0.06 | 0.12 | 0.21 | 0.05 | 0.11 | 0.01 | 0.00 | -0.01 |
| **A1** | 0.13 | 0.06 | -0.27 | -0.60 | -0.17 | 0.25 | 0.01 | 0.34 | -0.20 | 0.46 | -0.06 | 0.03 | -0.20 | -0.08 | -0.17 | -0.09 | 0.00 | -0.03 |
| **FA2G2** | -0.10 | -0.11 | -0.04 | 0.28 | 0.26 | 0.57 | 0.20 | -0.50 | -0.24 | 0.31 | -0.01 | -0.04 | -0.06 | -0.09 | -0.11 | -0.16 | 0.00 | -0.10 |
| **A3** | -0.02 | 0.01 | 0.04 | 0.04 | 0.11 | -0.22 | -0.06 | -0.05 | 0.06 | 0.26 | -0.48 | 0.65 | 0.38 | -0.06 | -0.24 | -0.03 | 0.00 | -0.09 |
| **FA1** | 0.02 | 0.06 | -0.30 | -0.51 | 0.65 | -0.10 | -0.08 | -0.31 | 0.04 | -0.31 | 0.00 | 0.00 | 0.00 | 0.00 | 0.00 | 0.00 | 0.00 | 0.00 |
| **FA2BG1** | 0.02 | 0.01 | 0.07 | 0.00 | 0.11 | 0.19 | -0.09 | 0.08 | -0.34 | 0.00 | -0.06 | 0.26 | -0.07 | 0.77 | 0.24 | 0.29 | 0.00 | 0.11 |
| **M5A1** | -0.02 | 0.04 | 0.00 | -0.06 | -0.17 | 0.29 | -0.17 | 0.01 | -0.14 | -0.32 | -0.72 | -0.31 | 0.17 | -0.11 | 0.16 | -0.10 | 0.00 | 0.18 |
| **FA1G1** | -0.02 | 0.05 | -0.05 | -0.13 | 0.03 | 0.24 | -0.18 | -0.01 | 0.45 | 0.35 | 0.17 | -0.18 | 0.58 | 0.12 | 0.39 | 0.01 | 0.00 | -0.07 |
| **FM5A1** | -0.02 | 0.01 | 0.02 | 0.02 | -0.03 | 0.18 | -0.10 | 0.00 | 0.47 | -0.09 | -0.10 | 0.34 | -0.50 | 0.09 | 0.31 | -0.50 | 0.00 | -0.06 |
| **M6A1** | -0.01 | 0.01 | 0.00 | 0.00 | -0.07 | 0.25 | -0.08 | 0.02 | 0.40 | -0.18 | -0.03 | -0.16 | 0.03 | 0.41 | -0.73 | 0.01 | 0.00 | -0.07 |
| **A2G2** | -0.02 | -0.01 | -0.02 | 0.01 | 0.02 | 0.23 | -0.02 | -0.04 | 0.33 | 0.03 | -0.17 | 0.10 | -0.30 | -0.29 | 0.06 | 0.79 | 0.00 | -0.05 |
| **FA2G1S1** | 0.00 | 0.00 | 0.00 | 0.00 | 0.00 | 0.00 | 0.00 | 0.00 | 0.00 | 0.00 | 0.00 | 0.00 | 0.00 | 0.00 | 0.00 | 0.00 | 1.00 | 0.00 |
| **FA2G2S1** | -0.01 | -0.01 | -0.01 | 0.01 | 0.04 | 0.08 | 0.01 | -0.04 | 0.13 | 0.09 | 0.13 | 0.14 | 0.03 | -0.09 | -0.10 | -0.03 | 0.00 | 0.95 |

Table S7: Diagonal matrix of singular values (**Σ**) obtained from SVD of reduced gain matrix **K**

| **σ1** | 6.22 | 0.00 | 0.00 | 0.00 | 0.00 | 0.00 | 0.00 | 0.00 | 0.00 | 0.00 | 0.00 | 0.00 | 0.00 | 0.00 | 0.00 |
| --- | --- | --- | --- | --- | --- | --- | --- | --- | --- | --- | --- | --- | --- | --- | --- |
| **σ2** | 0.00 | 3.68 | 0.00 | 0.00 | 0.00 | 0.00 | 0.00 | 0.00 | 0.00 | 0.00 | 0.00 | 0.00 | 0.00 | 0.00 | 0.00 |
| **σ3** | 0.00 | 0.00 | 2.21 | 0.00 | 0.00 | 0.00 | 0.00 | 0.00 | 0.00 | 0.00 | 0.00 | 0.00 | 0.00 | 0.00 | 0.00 |
| **σ4** | 0.00 | 0.00 | 0.00 | 0.80 | 0.00 | 0.00 | 0.00 | 0.00 | 0.00 | 0.00 | 0.00 | 0.00 | 0.00 | 0.00 | 0.00 |
| **σ5** | 0.00 | 0.00 | 0.00 | 0.00 | 0.61 | 0.00 | 0.00 | 0.00 | 0.00 | 0.00 | 0.00 | 0.00 | 0.00 | 0.00 | 0.00 |
| **σ6** | 0.00 | 0.00 | 0.00 | 0.00 | 0.00 | 0.45 | 0.00 | 0.00 | 0.00 | 0.00 | 0.00 | 0.00 | 0.00 | 0.00 | 0.00 |
| **σ7** | 0.00 | 0.00 | 0.00 | 0.00 | 0.00 | 0.00 | 0.41 | 0.00 | 0.00 | 0.00 | 0.00 | 0.00 | 0.00 | 0.00 | 0.00 |
| **σ8** | 0.00 | 0.00 | 0.00 | 0.00 | 0.00 | 0.00 | 0.00 | 0.31 | 0.00 | 0.00 | 0.00 | 0.00 | 0.00 | 0.00 | 0.00 |
| **σ9** | 0.00 | 0.00 | 0.00 | 0.00 | 0.00 | 0.00 | 0.00 | 0.00 | 0.10 | 0.00 | 0.00 | 0.00 | 0.00 | 0.00 | 0.00 |
| **σ10** | 0.00 | 0.00 | 0.00 | 0.00 | 0.00 | 0.00 | 0.00 | 0.00 | 0.00 | 0.05 | 0.00 | 0.00 | 0.00 | 0.00 | 0.00 |
| **σ11** | 0.00 | 0.00 | 0.00 | 0.00 | 0.00 | 0.00 | 0.00 | 0.00 | 0.00 | 0.00 | 0.00 | 0.00 | 0.00 | 0.00 | 0.00 |
| **σ12** | 0.00 | 0.00 | 0.00 | 0.00 | 0.00 | 0.00 | 0.00 | 0.00 | 0.00 | 0.00 | 0.00 | 0.00 | 0.00 | 0.00 | 0.00 |
| **σ13** | 0.00 | 0.00 | 0.00 | 0.00 | 0.00 | 0.00 | 0.00 | 0.00 | 0.00 | 0.00 | 0.00 | 0.00 | 0.00 | 0.00 | 0.00 |
| **σ14** | 0.00 | 0.00 | 0.00 | 0.00 | 0.00 | 0.00 | 0.00 | 0.00 | 0.00 | 0.00 | 0.00 | 0.00 | 0.00 | 0.00 | 0.00 |
| **σ15** | 0.00 | 0.00 | 0.00 | 0.00 | 0.00 | 0.00 | 0.00 | 0.00 | 0.00 | 0.00 | 0.00 | 0.00 | 0.00 | 0.00 | 0.00 |
| **σ16** | 0.00 | 0.00 | 0.00 | 0.00 | 0.00 | 0.00 | 0.00 | 0.00 | 0.00 | 0.00 | 0.00 | 0.00 | 0.00 | 0.00 | 0.00 |
| **σ17** | 0.00 | 0.00 | 0.00 | 0.00 | 0.00 | 0.00 | 0.00 | 0.00 | 0.00 | 0.00 | 0.00 | 0.00 | 0.00 | 0.00 | 0.00 |
| **σ18** | 0.00 | 0.00 | 0.00 | 0.00 | 0.00 | 0.00 | 0.00 | 0.00 | 0.00 | 0.00 | 0.00 | 0.00 | 0.00 | 0.00 | 0.00 |

Table S8: Unitary matrix **V^T^** obtained from SVD of reduced gain matrix **K**

|  | **MnCl_2_** | **EDTA** | **Mn-EDTA** | **Mn T1** | **Mn T2** | **ED T1** | **ED T2** | **Mn-ED T2** | **ED-Mn T1** | **Mn T1 - ED T1** | **Mn T2 - ED T1** | **Mn T2 - ED T2** | **EDTA - Mn T1 - ED T1** | **EDTA - Mn T2 - ED T1** | **EDTA - Mn T2 - ED T2** |
| --- | --- | --- | --- | --- | --- | --- | --- | --- | --- | --- | --- | --- | --- | --- | --- |
| **μ1** | -0.39 | 0.44 | -0.58 | 0.25 | 0.11 | -0.38 | 0.18 | 0.00 | 0.09 | 0.09 | -0.07 | 0.11 | -0.09 | 0.07 | -0.11 |
| **μ2** | 0.60 | 0.13 | 0.24 | 0.03 | -0.21 | -0.47 | 0.35 | 0.00 | -0.07 | 0.18 | -0.13 | 0.21 | -0.18 | 0.13 | -0.21 |
| **μ3** | 0.00 | 0.65 | 0.45 | 0.42 | -0.16 | 0.10 | -0.28 | -0.02 | 0.20 | -0.08 | 0.06 | -0.09 | 0.08 | -0.06 | 0.09 |
| **μ4** | -0.18 | 0.11 | 0.18 | -0.08 | 0.25 | 0.27 | -0.34 | -0.10 | -0.30 | 0.31 | -0.23 | 0.36 | -0.31 | 0.23 | -0.36 |
| **μ5** | -0.39 | -0.34 | 0.33 | 0.57 | -0.05 | 0.11 | 0.48 | 0.17 | -0.15 | 0.04 | -0.03 | 0.05 | -0.04 | 0.03 | -0.05 |
| **μ6** | 0.49 | -0.06 | -0.48 | 0.48 | -0.09 | 0.52 | -0.03 | -0.04 | 0.01 | 0.05 | -0.04 | 0.06 | -0.05 | 0.04 | -0.06 |
| **μ7** | 0.26 | 0.09 | 0.12 | 0.13 | 0.91 | -0.02 | 0.16 | 0.03 | -0.03 | -0.08 | 0.06 | -0.09 | 0.08 | -0.06 | 0.09 |
| **μ8** | -0.01 | -0.28 | 0.08 | 0.04 | 0.12 | -0.05 | -0.13 | 0.16 | 0.87 | 0.13 | -0.10 | 0.15 | -0.13 | 0.10 | -0.15 |
| **μ9** | -0.05 | 0.37 | 0.03 | -0.41 | -0.04 | 0.52 | 0.60 | 0.06 | 0.23 | 0.03 | -0.02 | 0.03 | -0.03 | 0.02 | -0.03 |
| **μ10** | -0.06 | -0.11 | 0.07 | 0.07 | 0.02 | -0.01 | 0.15 | -0.96 | 0.16 | -0.01 | 0.00 | -0.01 | 0.01 | 0.00 | 0.01 |
| **μ11** | 0.00 | 0.00 | 0.00 | 0.00 | 0.00 | 0.00 | 0.00 | 0.00 | 0.00 | -0.16 | 0.46 | 0.49 | 0.27 | -0.46 | -0.49 |
| **μ12** | 0.00 | 0.00 | 0.00 | 0.00 | 0.00 | 0.00 | 0.00 | 0.00 | 0.00 | -0.52 | -0.43 | 0.18 | 0.54 | 0.43 | -0.18 |
| **μ13** | 0.00 | 0.00 | 0.00 | 0.00 | 0.00 | 0.00 | 0.00 | 0.00 | 0.00 | 0.73 | -0.03 | -0.04 | 0.68 | 0.03 | 0.04 |
| **μ14** | 0.00 | 0.00 | 0.00 | 0.00 | 0.00 | 0.00 | 0.00 | 0.00 | 0.00 | 0.00 | 0.54 | -0.45 | 0.00 | 0.54 | -0.45 |
| **μ15** | 0.00 | 0.00 | 0.00 | 0.00 | 0.00 | 0.00 | 0.00 | 0.00 | 0.00 | 0.00 | -0.45 | -0.54 | 0.00 | -0.45 | -0.54 |

**Supplementary Information S4**

**Dynamic glycosylation model:** One may acquire a mechanistic understanding of the effect of media supplementation on the glycan distribution profile either experimentally, or via simulation using mathematical models. Several mathematical models have been proposed for predicting glycan distribution profiles in mAbs (del Val et al. 2011; Hossler et al. 2007; Jedrzejewski et al. 2014; Krambeck and Betenbaugh 2005). In this work, we used an established dynamic kinetic model (del Val et al. 2011) to test the proposition that the changes observed in the glycan distribution due to the addition of MnCl_2_ are induced by changes in the concentrations of the glycosyltransferase enzymes. First, we defined a glycan reaction network by specifying a set of glycan reaction rules and generated a network consisting of 16 glycan species participating in 20 reactions. The reaction rules and the associated kinetic constants for each enzyme participating in the reaction network are listed in Table S9. Next, by assuming that the Golgi compartment can be modeled as a plug flow reactor (PFR) based on the cisternal maturation model (del Val et al. 2011), we obtained material balances for each of these 16 glycan species. The resulting system of partial differential equations was solved using the *ode15s* function in MATLAB to obtain the dynamic concentration profile for each of the sixteen glycan species. We then estimated the kinetic parameters for the enzyme activities and the maximum enzyme concentration using the optimization routine *fminsearch* in MATLAB, and the resulting kinetic model was used to assess the effect of adding MnCl_2_ on D0 exerts on the final glycan distribution.

Figure S1 shows a comparison of the glycan distribution predicted by the adapted kinetic model and corresponding experimental glycan distribution data from two of the tested conditions – control and MnCl_2_ supplementation on D0. First we matched the simulated glycan profile to the experimentally determined glycan profile in the control flask using kinetic parameters obtained by the optimization subroutine. Next, we note that the addition of MnCl_2_ on D0 of the cell culture results in a decrease in the relative abundance of fucosylated glycoforms, FA2 and FA2G1 with a corresponding increase in the concentration of their afucosylated isoforms, A2 and A2G1 species. The enzyme alpha-1,6-fucosyltransferase (FucT) is responsible for fucosylating glycan species in the N-glycan biosynthetic pathway. By reducing the total enzyme concentration for FucT in the kinetic model from 0.55 mM to 0.22 mM, we are able to simulate the change in the glycan distribution due to addition of MnCl_2_ on D0, as shown in Figure S1. b.

Table S9: Reaction rules for generating the glycan reaction network used in simulations of the dynamic glycosylation model

| **Enzyme** | **Co-substrate** | **Rule** | **Reaction** | **k_f_, min^-1^** | **K_m_, mM** | **K_md_, mM** |
| --- | --- | --- | --- | --- | --- | --- |
| ManI | Water | Man > 8 | Man = Man-1; | 888 | 60.5 | 0 |
| ManI | Water | Man > 7 && ~(Man>8) | Man = Man-1; | 888 | 5 | 0 |
| ManI | Water | Man > 6 && ~(Man>7) | Man = Man-1; | 888 | 5 | 0 |
| ManI | Water | Man > 5 && ~(Man>6) | Man = Man-1; | 888 | 5 | 0 |
| ManII | Water | Man>3 && Br4==1 && Gnb==0 && Man~=4 | Man = Man-1; | 1924 | 200 | 0 |
| FucT | GDP_Fuc | Fuc==0 && Br4>0 && Gnb==0 && Gal==0 && Man==3 | Fuc = Fuc+1; | 291 | 5 | 46 |
| GnTI | UDP_Gn | Br4==0 && Man==5 | Br4 = Br4+1; | 1022 | 260 | 170 |
| GnTII | UDP_Gn | Br2==0 && Man<4 && Br4==1 && Gnb==0 | Br2 = Br2+1; | 50 | 20 | 960 |
| GalT | UDP_Gal | (Br2==1) && ~(Gnb>0 && Br2>0) && Man<4 | Br2 = Br2+1; Gal = Gal+1; | 872 | 135 | 65 |
| GalT | UDP_Gal | (Br4==1) && ~(Gnb>0 && Br2>0) && Man<4 | Br4 = Br4+1; Gal = Gal+1; | 872 | 135 | 65 |
| ManII | Water | Man>3 && Br4==1 && Gnb==0 && Man==4 | Man = Man-1; | 1924 | 100 | 0 |
| GalT | UDP_Gal | (Br2==1 && Br4 ==1) && (Gnb>0 && Br2>0) | Br2 = Br2+1; Gal = Gal+1; | 872 | 488.7 | 65 |
| GalT | UDP_Gal | (Br4==1 && Br4==1) && (Gnb>0 && Br2>0) | Br4 = Br4+1; Gal = Gal+1; | 872 | 6280 | 65 |

Table S10: Enzyme and co-substrate concentrations used in the dynamic glycosylation model

| Enzyme | [E_0_], μM | |  |
| --- | --- | --- | --- |
| FucT | 0.55 | |  |
| GalT | 2.06 | |  |
| GnTI | 9.66 | |  |
| GnTII | 3.96 | |  |
| ManI | 9.09 | |  |
| ManII | 2.85 | |  |
| Cosubstrate | | Conc., mM | |
| GDP_Fuc | | 576 | |
| UDP_Gal | | 265 | |
| UDP_Gn | | 1057 | |
| Water | | 1 | |

**
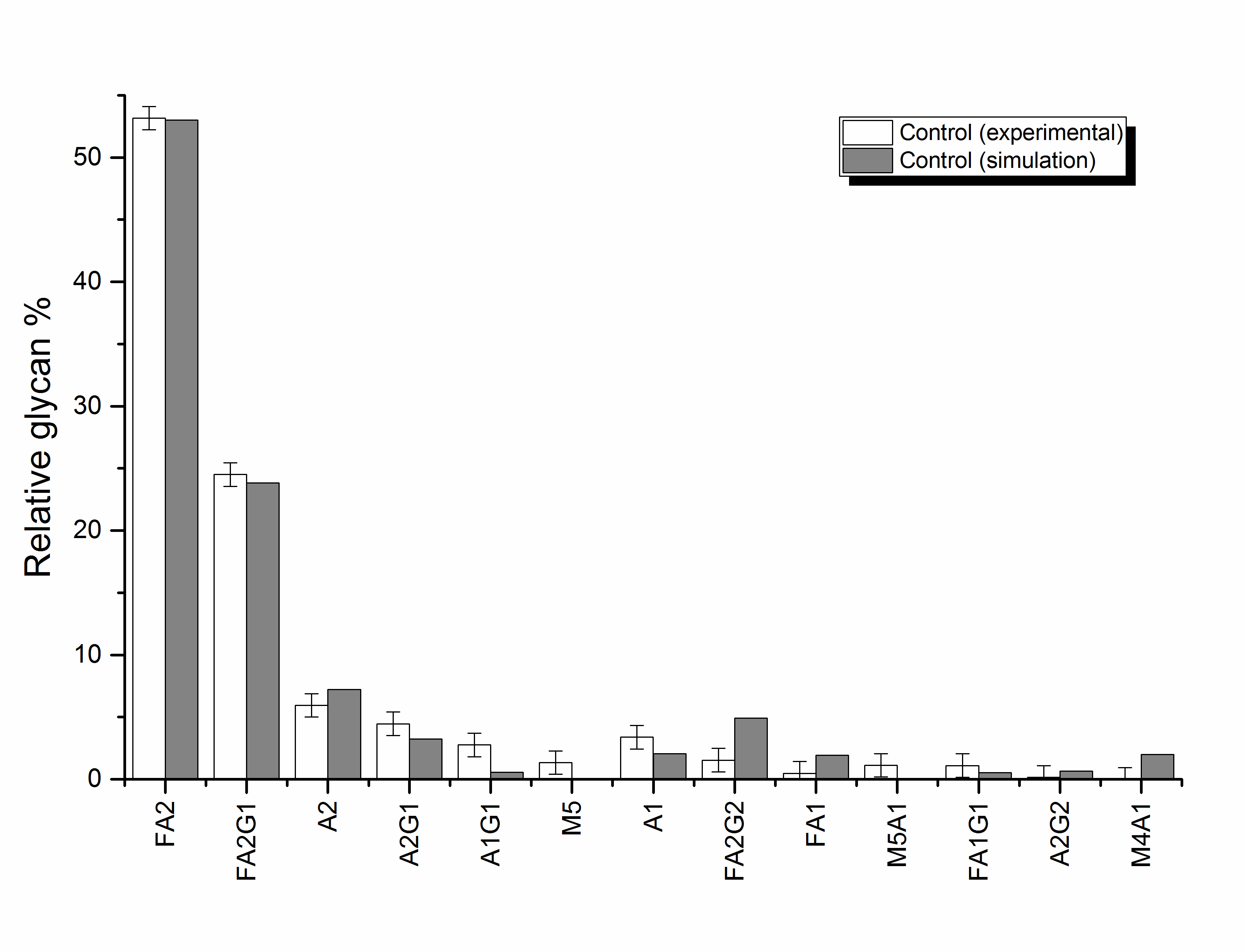

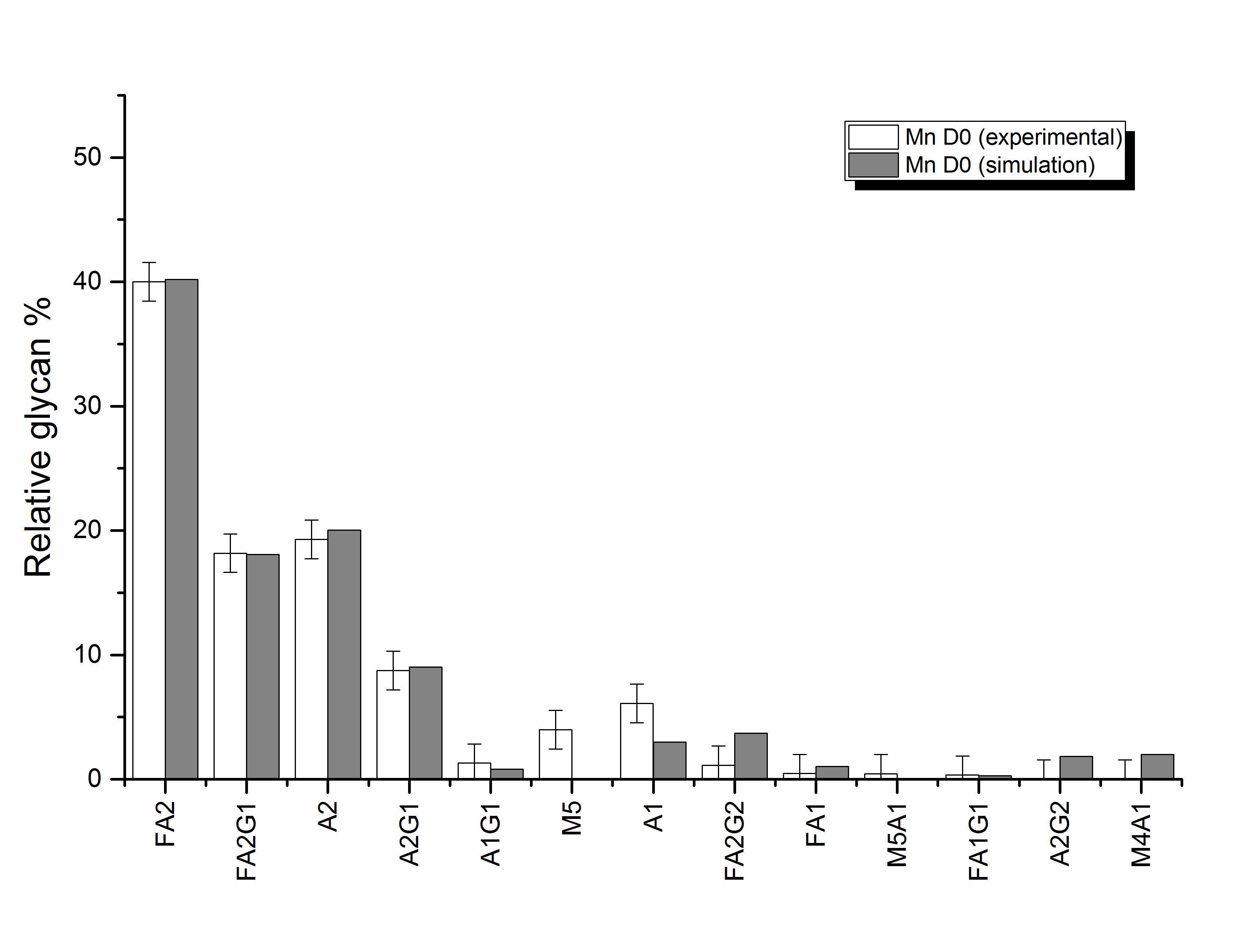
**

(a)

(b)

Figure S1: Comparison of experimental data and model fit for the glycan distribution profile obtained from (a) control flask; and (b) when MnCl_2_ is added on D0.

**Supplementary Information S5**

(b)

(a)

(c)

(d)

Figure S2: Normalized glucose concentration data for each condition tested, i.e. when (a) EDTA is added by itself on D0, or in the presence of MnCl_2_ on D0, D3, and D6; (b) EDTA is added by itself on D3, or in the presence of MnCl_2_ on D0, D3, and D6; (c) EDTA is added by itself on D6, or in the presence of MnCl_2_ on D0, D3, and D6; and (d) MnCl_2_ is added on D0, D3, and D6.

**Supplementary Information S6**

| **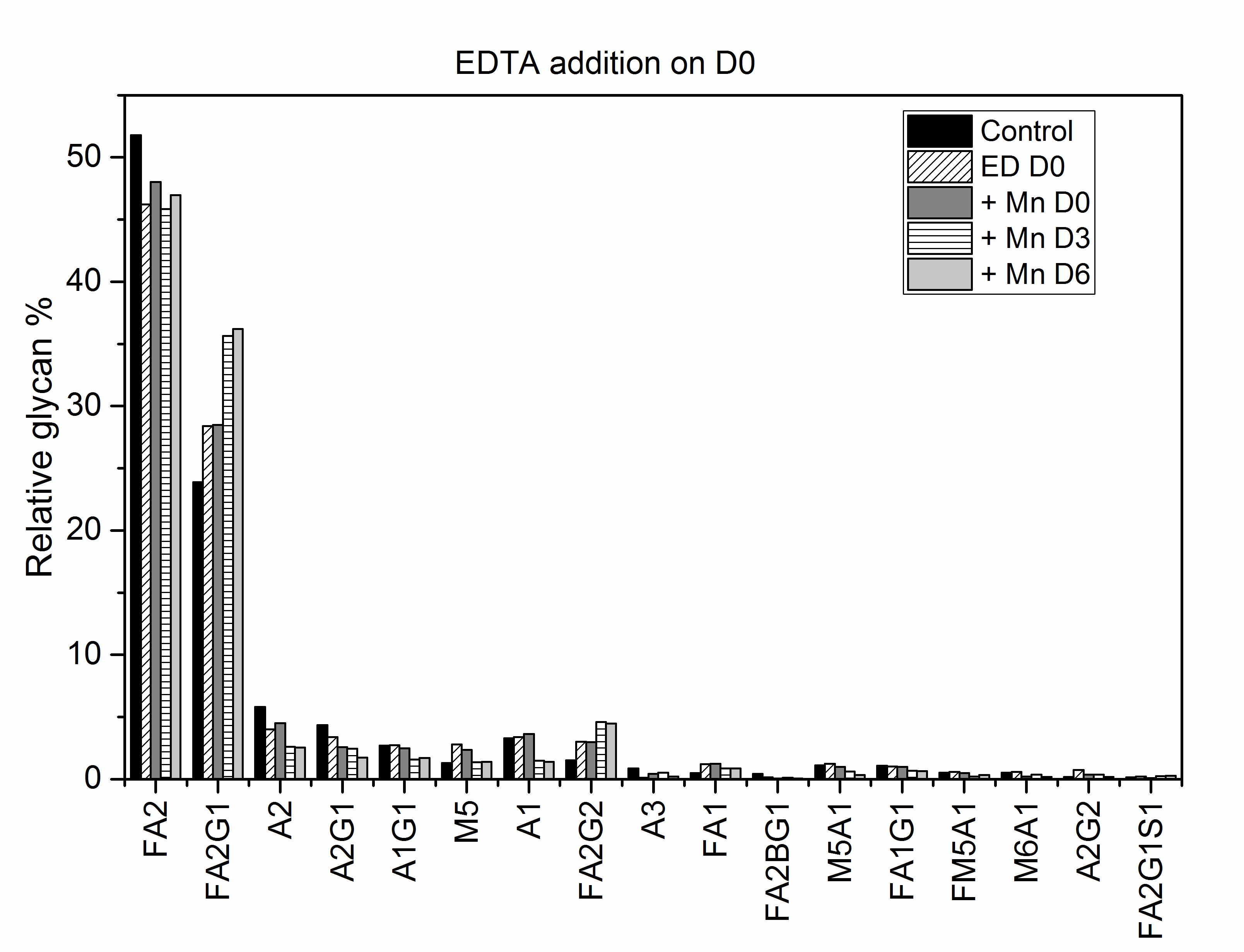**  (**a**) | **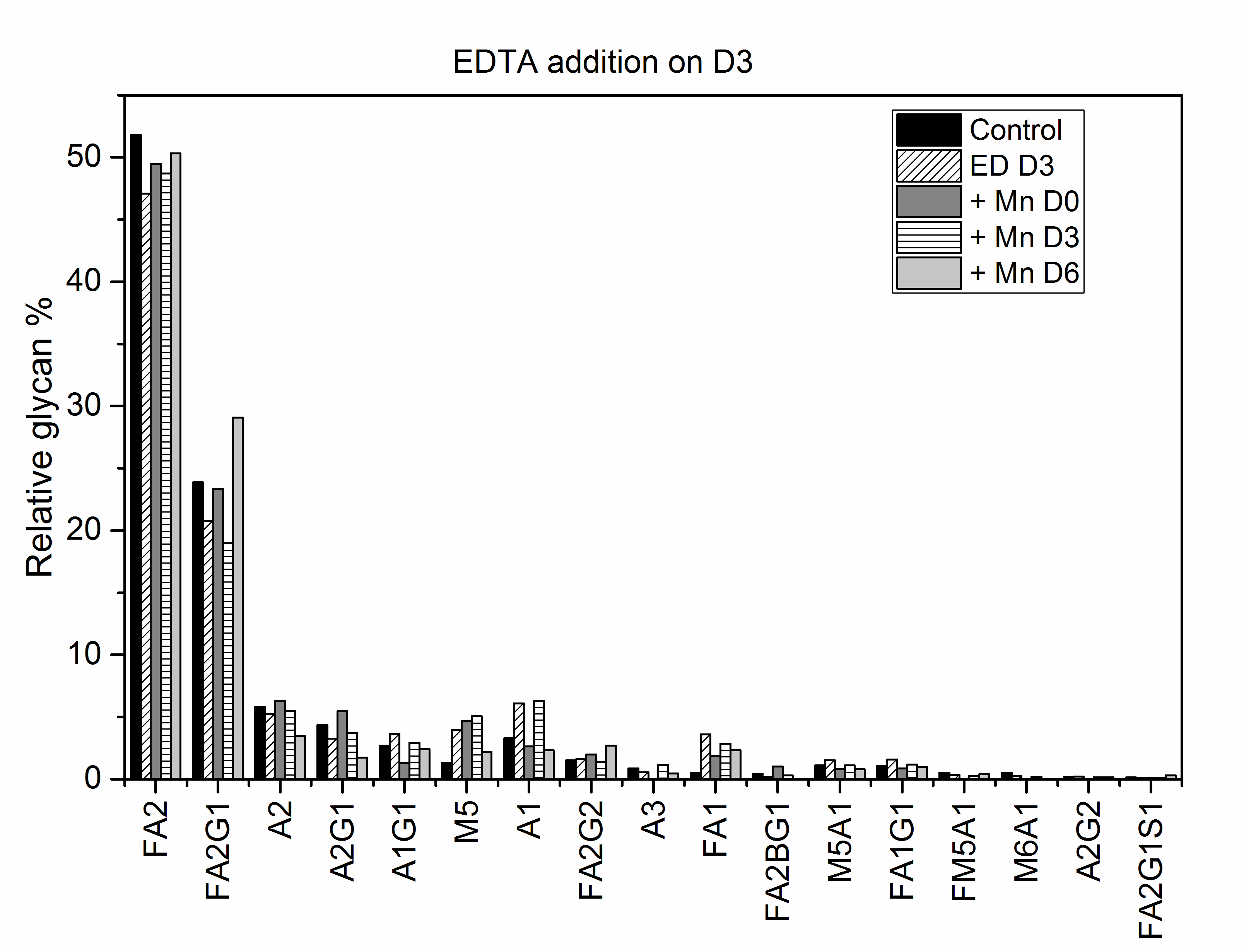**  (**b**) |
| --- | --- |
| **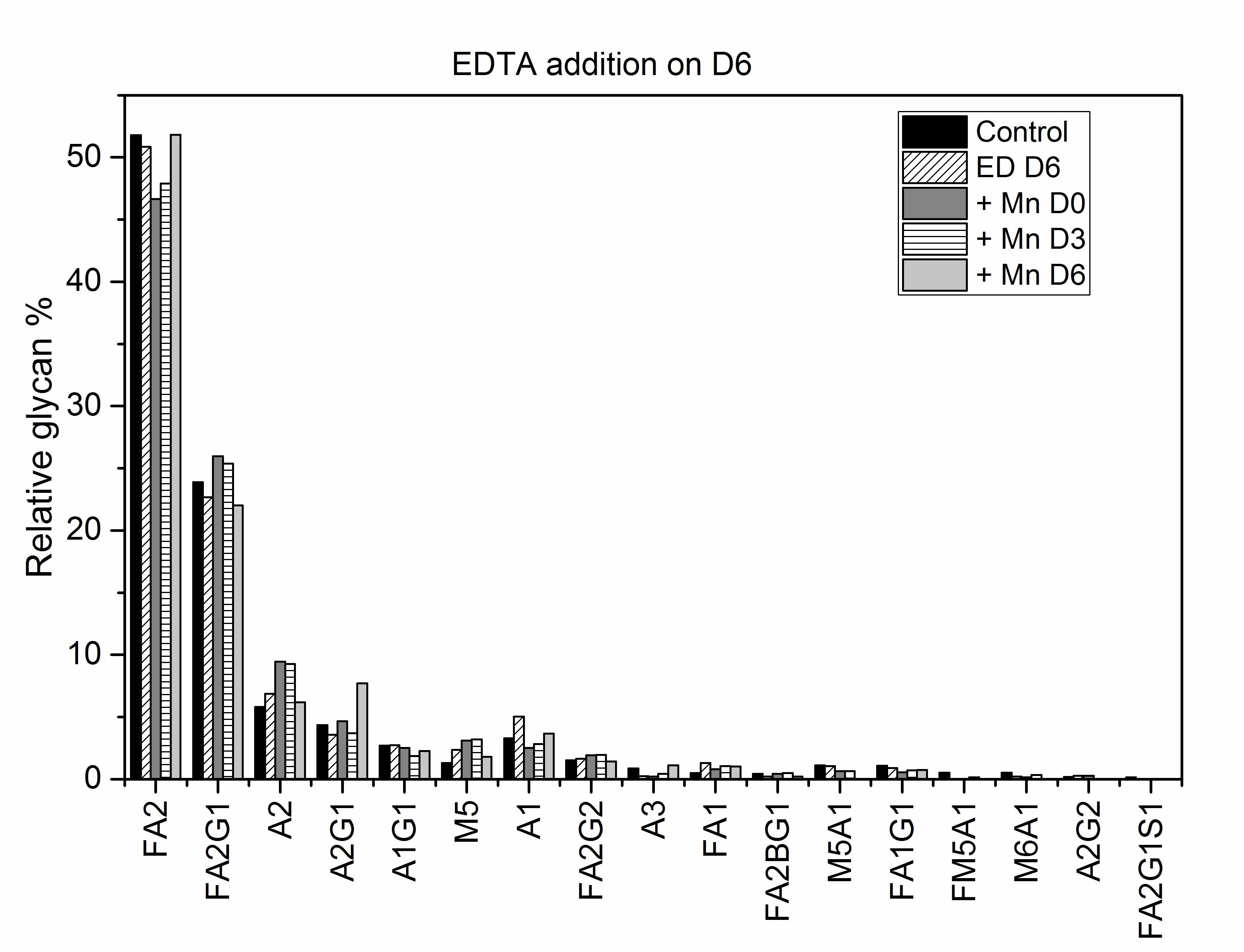**  (**c**) | **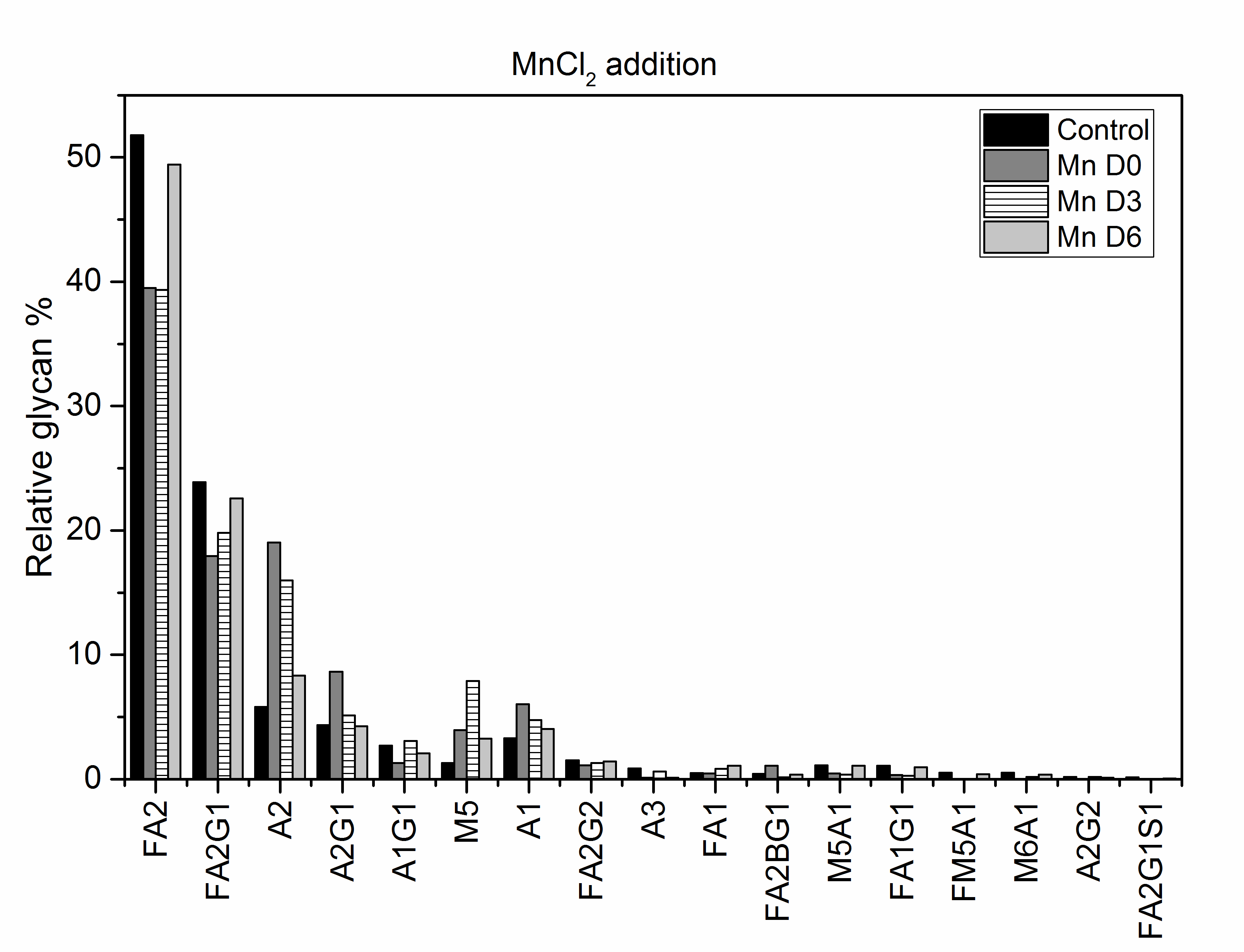**  (**d**) |

Figure S3: Average relative glycan percentage of IgG1 glycans produced in CHO-K1 cells when: (a) EDTA is added on D0 with no MnCl_2_ supplementation or with MnCl_2_ supplementation on D0, D3, and D6; (b) EDTA added on D3 with no MnCl_2_ supplementation or with MnCl_2_ supplementation on D0, D3, and D6; (c) EDTA added on D6 with no MnCl_2_ supplementation or with MnCl_2_ supplementation on D0, D3, and D6; and (d) MnCl_2_ is added on D0, D3, and D6**.**

**Supplementary Information S7**


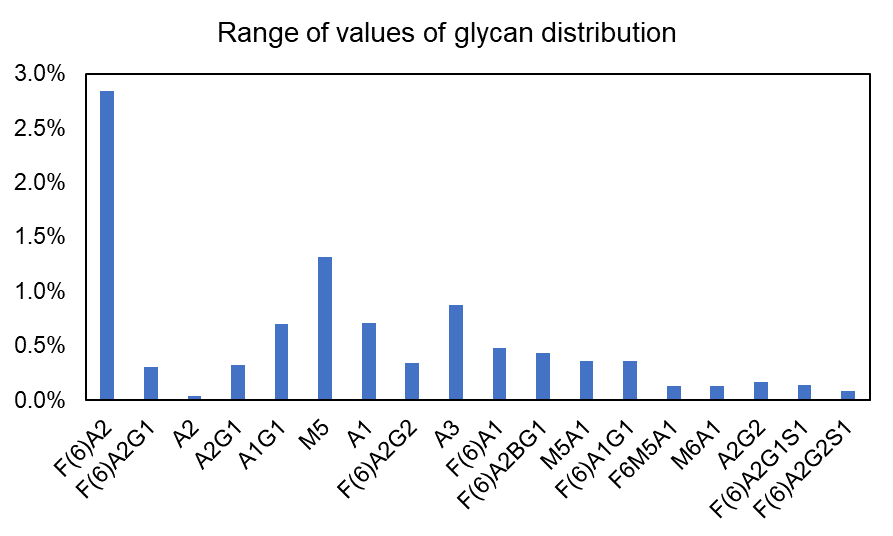


Figure S4: Normal range of values for glycan concentration obtained from the control samples.

**References**

del Val IJ, Nagy JM, Kontoravdi C. 2011. A dynamic mathematical model for monoclonal antibody N-linked glycosylation and nucleotide sugar donor transport within a maturing Golgi apparatus. Biotechnology Progress 27(6):1730-1743.

Hossler P, Mulukutla BC, Hu WS. 2007. Systems Analysis of N-Glycan Processing in Mammalian Cells. Plos One 2(8).

Jedrzejewski PM, del Val IJ, Constantinou A, Dell A, Haslam SM, Polizzi KM, Kontoravdi C. 2014. Towards Controlling the Glycoform: A Model Framework Linking Extracellular Metabolites to Antibody Glycosylation. International Journal of Molecular Sciences 15(3):4492-4522.

Krambeck FJ, Betenbaugh MJ. 2005. A mathematical model of N-linked glycosylation. Biotechnology and Bioengineering 92(6):711-728.

McDonald A, Tipton K, Stroop C, Davey G. 2010. GlycoForm and Glycologue: two software applications for the rapid construction and display of N-glycans from mammalian sources. BMC Research Notes 3(1):173.
